# Target-oriented prioritization: targeted selection strategy by integrating organismal and molecular traits through predictive analytics in breeding

**DOI:** 10.1186/s13059-022-02650-w

**Published:** 2022-03-15

**Authors:** Wenyu Yang, Tingting Guo, Jingyun Luo, Ruyang Zhang, Jiuran Zhao, Marilyn L. Warburton, Yingjie Xiao, Jianbing Yan

**Affiliations:** 1 National Key Laboratory of Crop Genetic Improvement, Huazhong Agricultural University, Wuhan, 430070 China; 2 College of Science, Huazhong Agricultural University, Wuhan, 430070 China; 3 Hubei Hongshan Laboratory, Wuhan, 430070 China; 4 Beijing Key Laboratory of Maize DNA Fingerprinting and Molecular Breeding, Beijing Academy of Agricultural & Forestry Sciences, Beijing, 100097 China; 5 United States Department of Agriculture-Agricultural Research Service, Corn Host Plant Resistance Research Unit, Box 9555, Mississippi State, MS 39762 USA

**Keywords:** Crop breeding, Multiple traits, Genomic prediction, Omics, Machine learning

## Abstract

Genomic prediction in crop breeding is hindered by modeling on limited phenotypic traits. We propose an integrative multi-trait breeding strategy via machine learning algorithm, target-oriented prioritization (TOP). Using a large hybrid maize population, we demonstrate that the accuracy for identifying a candidate that is phenotypically closest to an ideotype, or target variety, achieves up to 91%. The strength of TOP is enhanced when omics level traits are included. We show that TOP enables selection of inbreds or hybrids that outperform existing commercial varieties. It improves multiple traits and accurately identifies improved candidates for new varieties, which will greatly influence breeding.

**Supplementary Information:**

The online version contains supplementary material available at 10.1186/s13059-022-02650-w.

## Background

The likelihood of reduced yields of major food crops due to the changing climate continues to rise, as does the global population, thus making the development of genetically improved, climate-resilient crops a research priority [1–3]. Conventional breeding approaches have made enormous contributions to increased food production, but the current pace of improvement is insufficient to meet future demands [4, 5]. New plant breeding techniques are needed to contribute to accelerated improvements in crop productivity and sustainability [3, 6, 7].

Genomics, phenomics, and analytics technologies are powerful tools to boost breeding progress [7, 8]. Jointly, they allow genomic prediction to capitalize on the genotype-phenotype relationships at the whole-genome level. Genomic prediction and selection have been implemented in many crops to accelerate the breeding process in public and private breeding programs [9–11]. Although genomic prediction has successfully increased genetic gain in numerous programs [12, 13], it may be possible to develop new crop varieties more effectively and reach greater genetic gains with emerging technologies. The effect of selection on multiple traits simultaneously, incorporation of omics data, and application of machine learning algorithms on the efficiency of genomic prediction has not been fully elucidated.

Selecting individuals that are higher yielding, resistant to stress and disease, or otherwise more attractive is of extreme consequence in plant breeding and has been since the first farmers began the process of plant domestication [14]. One significant change during modern maize breeding was the creation of phenotypes suited for growing in a diverse set of agronomic environments, including adaptation to high planting density [15]. Plant breeders are usually interested in improving multiple traits, but breeding for two or more traits simultaneously is generally more difficult than breeding for a single trait [16]. Three basic procedures for carrying out multi-trait selection have been described: tandem selection, independent culling levels, and index selection. Index selection is expected to be more efficient than tandem selection, independent culling levels [17] (Additional file 1: Fig. S1). A base index has been utilized in genomic prediction for selecting individuals on an array of genomic-predicted values for multiple traits; however, the desired selection index must be created for each specific population and breeding goal. Progress has also been made on multivariate genomic selection [18, 19] and crop growth models [20, 21]; these methods dissect yield into traits measured at high throughput and at an early stage to achieve selection accuracy and logistical efficiency. However, genomic prediction can be advanced by shifting the focus from single traits to the identification of individual plants that come closest to an ideotype, or target variety, which combines merits from multiple traits.

We applied omics and machine learning towards the goal of correctly identifying the best breeding candidates. Post-genomics technologies including transcriptomes, proteomics, and metabolomics offer mechanistic links between genotype and phenotype [22]. Together with genomics, these other omics data offer opportunities for the comprehensive and systematic analysis of biological discoveries [23, 24], and performance prediction in inbred and hybrid breeding [11]. Machine learning has been broadly applied to analyze omics datasets to understand functional multi-omics relationships, and to discover novel variants [25]; however, it has rarely been used in selecting breeding varieties.

**Fig. 1:**
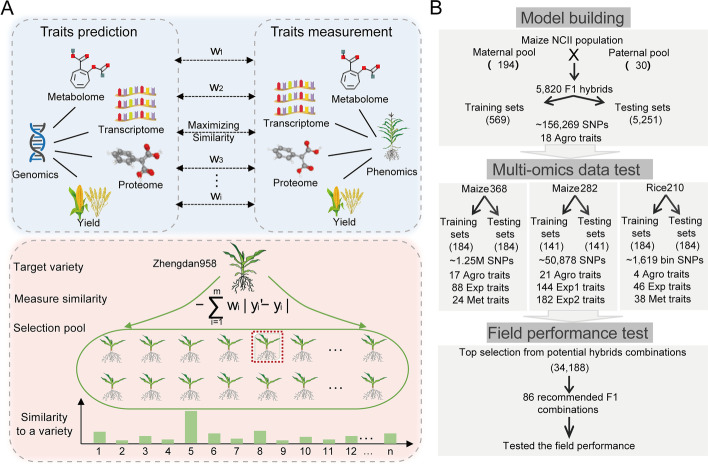
Multiple selection schemes in crop breeding. **A** The schematic workflow of the TOP algorithm. By learning the optimal trait weights using the maximum likelihood algorithm, genomic predictions of multiple traits are integrated to select the best individual candidates from diverse breeding pools, maximizing the global similarity to an ideotype or target. **B** Flowchart illustrating the process of model building, multi-omics data test, and field performance test in the present study

Here we present an integrative multi-trait breeding strategy that incorporates agronomic and omics traits (transcriptomes and metabolomes) to predict the best performing candidates to create new varieties through a machine learning algorithm. This algorithm, called target-oriented prioritization (TOP), learns the inherent correlations among traits in a training population, balances the selection of multiple traits simultaneously, and predicts the degree of similarity between an untested genotype and a target, which can be a current commercial variety (Fig. 1A). We examined this strategy in a maize NCII population and calculated the accuracy of identifying a breeding candidate of a predefined target. This strategy was further extrapolated to two independent maize populations of diverse inbred lines and a rice population of recombinant inbred lines. We discuss how an integrative multi-trait breeding strategy can be utilized for selecting hybrids that outperform a current commercial variety in breeding practices (Fig. 1B).

## Results

### Genomic prediction for individual traits in a maize NCII population

**Fig. 2:**
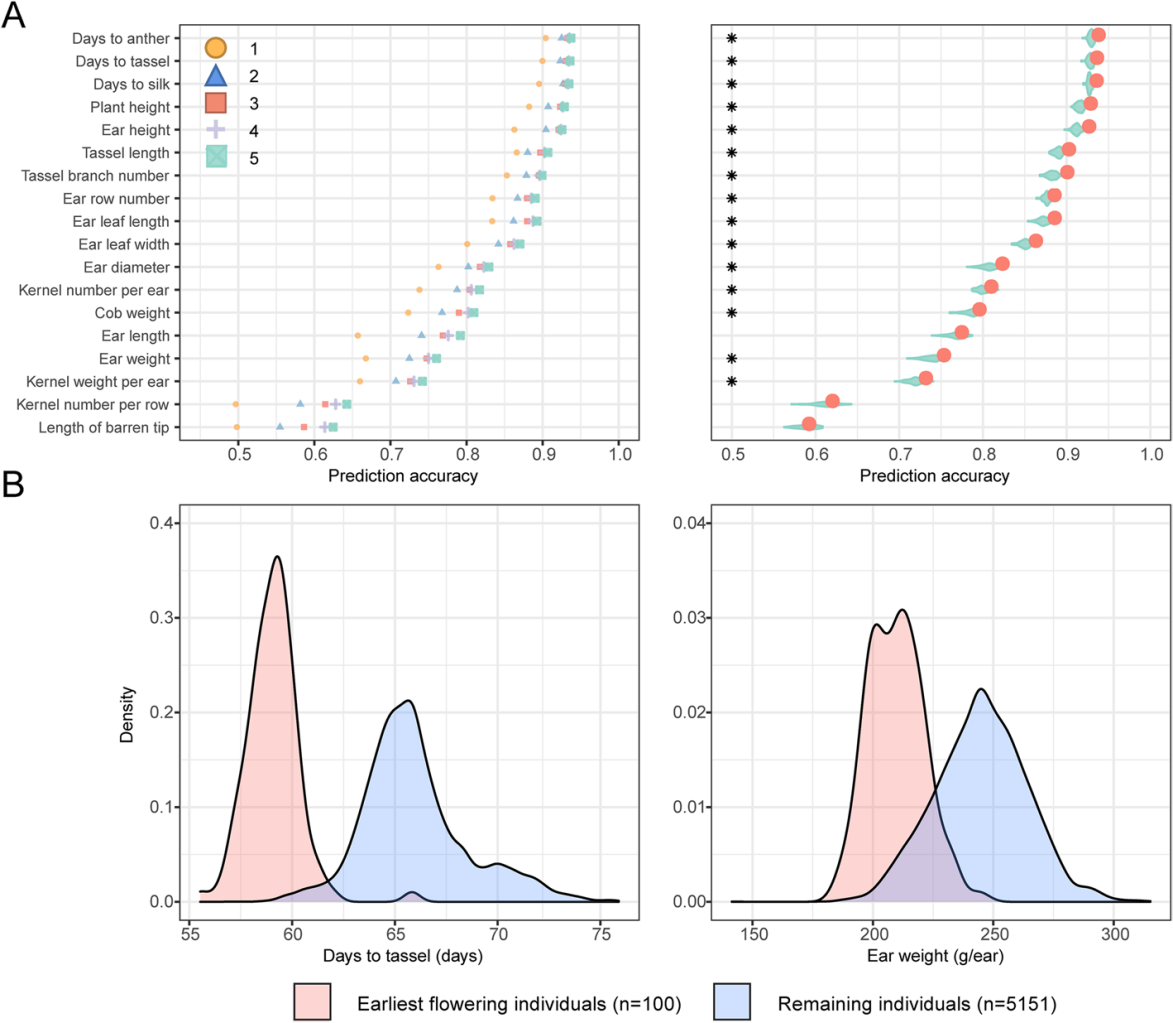
Genomic prediction of agronomic traits in a maize NCII population. **A** The performance of prediction accuracy by different training datasets and the prediction accuracy between specifically and randomly selected training datasets. In the 5820 F_1_ hybrids with 194 maternal and 30 paternal lines, the F_1_s of 1 to 5 diagonal strips were chosen to be the training set, and the remaining F_1_s acted as the testing set (see Supplementary Fig. 1 for details). The prediction accuracy is evaluated by the Pearson correlation coefficient (*r*) between the prediction and measured phenotype in the testing set. The red dot indicates the prediction accuracy using three-diagonal strips as the training set (569 individuals). The violin plot indicates the prediction accuracy of 569 randomly selected individuals used as training sets that are repeated 100 times. The significant difference between three-diagonal F_1_s and random F_1_s used as training sets is marked by an asterisk for each trait (*P*<0.01, Student’s *t* test). **B** Trait performances achieved by selecting the earliest flowering F_1_ hybrids. From the testing set of 5251 F_1_ hybrids, the 100 earliest flowering individuals were selected based on genomic prediction of days to tassel. The early flowering-based selection (red) were compared to the remaining individuals (blue) for days to tassel and ear weight, based on Student’s *t* test (*P*<0.01)

We first evaluated genomic prediction accuracy for 18 individual traits by applying a mixed linear model in a maize NCII population of 5820 F_1_ hybrids with 156,269 SNPs (see the “Methods” section). The maize NCII population consists of 194 maternal inbred lines and 30 paternal lines originating from different heterotic groups. Genomic prediction was conducted with a training set composed of hybrids in the leading diagonal line of the NCII mating scheme (Additional file 1: Fig. S2), which is expected to improve trait prediction accuracy, the Pearson correlation coefficient between predicted and observed values, by maximizing connectedness between training set and testing set (composed of the remaining, off-diagonal hybrids). As a result, the prediction accuracy was moderate to high for all traits, ranging from 0.5 to 0.9 (Fig. 2A left panel).

Prediction accuracy increased when more hybrids from adjacent diagonal strips were included in the training set (Fig. 2A left panel) but showed no further significant improvement after the training set exceeded 569 hybrids (3 diagonal strips). Compared to a random sample of equal size, this 569-hybrid training set exhibited significantly higher accuracy (*P*<0.01, Fig. 2A right panel) for most traits and hereafter was used as the training set for examining selection accuracy and exploring the integrative multi-trait breeding strategy.

To mimic the selection based on genomic prediction, we assessed the actual phenotypic difference between selected and unselected hybrids for one trait by comparing the top 100 hybrids to those remaining in the testing set, in which the hybrids were listed in descending order of favorability by genomic-predicted values. As a result, the top 100 hybrids selected for flowering time indeed flowered 6 days earlier than others on average (*P*=3.68E−81) but showed a significant loss of ear weight (35 g, *P*=4.12E−55) (Fig. 2B). This nonsynergistic selection, i.e., selection benefiting one trait but not others, was often observed when performing the one-trait directed selection for pairs of traits (Additional file 1: Fig. S3) and would cause difficulty in the selection of breeding materials.

### Integrative multi-trait breeding strategy and its application in a maize NCII population

To address the inefficiencies presented by the selection via single-trait genomic prediction, we proposed a machine learning algorithm to integrate genomic predictions from multiple traits for selecting individual candidates that are systematically similar to an ideotype. We call this algorithm TOP, or target-oriented prioritization. There are two key steps in this implementation: (1) learning multi-trait similarity between genomic-predicted and field-observed values for obtaining optimal weights, which represent the balanced status of individual traits in maximizing the overall performance and similarity to the ideotype, and (2) predicting the multi-trait similarity between predicted objects (inbreds or hybrids) and a target (see the “Methods” section and Fig. 1A). Unlike the prediction accuracy used in single-trait genomic prediction, the selection accuracy of TOP is defined as the identification rate that is high if the target is in a candidate pool of genotypes and the algorithm can identify it (see the “Methods” section).

We first considered only three flowering time traits in the maize NCII population. TOP identified the target from a pool containing 20 candidate hybrids with a rate of 0.322, but the identification rate decreased rapidly as the pool size increased (Table 1). The six plant architecture traits enabled to identify the target from the rate of 0.801 in 20 hybrids, indicating that different trait types contributed different identification abilities. We next integrated all 18 agronomic traits into TOP, and the identification rate increased considerably (for example, to 0.909 in the pool of 20 hybrids and 0.686 in the pool of 200 hybrids; Table 1). When TOP was compared to random identification, its advantage was enhanced exponentially with increasing pool size. By combining the information from many phenotypes, the integrative multi-trait breeding strategy or TOP has great potential for identifying improved varieties and will be particularly helpful in large-scale breeding programs.

**Table 1:** The identification rate of TOP algorithm in the maize NCII popualtion

Pool size^**a**^	Flowering time (3)^**b**^	Plant architecture (6)	Flowering time +Plant architecture (9)	Yield (9)	Full (18)	Random^**c**^	Improvement^**d**^
**20**	0.322	0.801	0.844	0.545	0.909	0.05	18.2
**50**	0.188	0.681	0.736	0.392	0.841	0.02	42.1
**100**	0.116	0.571	0.636	0.293	0.771	0.01	77.1
**200**	0.069	0.454	0.526	0.212	0.686	0.005	137.2
**400**	0.039	0.345	0.416	0.148	0.587	0.0025	234.8
**600**	0.028	0.287	0.355	0.117	0.529	0.00167	316.8
**800**	0.022	0.248	0.314	0.098	0.486	0.00125	388.8
**1000**	0.018	0.222	0.283	0.086	0.453	0.001	453.0

^a^Pool size indicates the number of candidate individuals needed to be searched for the target

^b^The trait group that used to identify the candidate individual similar to a target, the value within the parenthesis indicates the number of traits at this group

^c^It indicates the probability of successful identification of a defined target from a *N*-sized pool by chance.

^d^It indicates the fold of the TOP accuracy beyond the random, equal to the identification rate using 18 traits divided by the value by chance

**Fig. 3:**
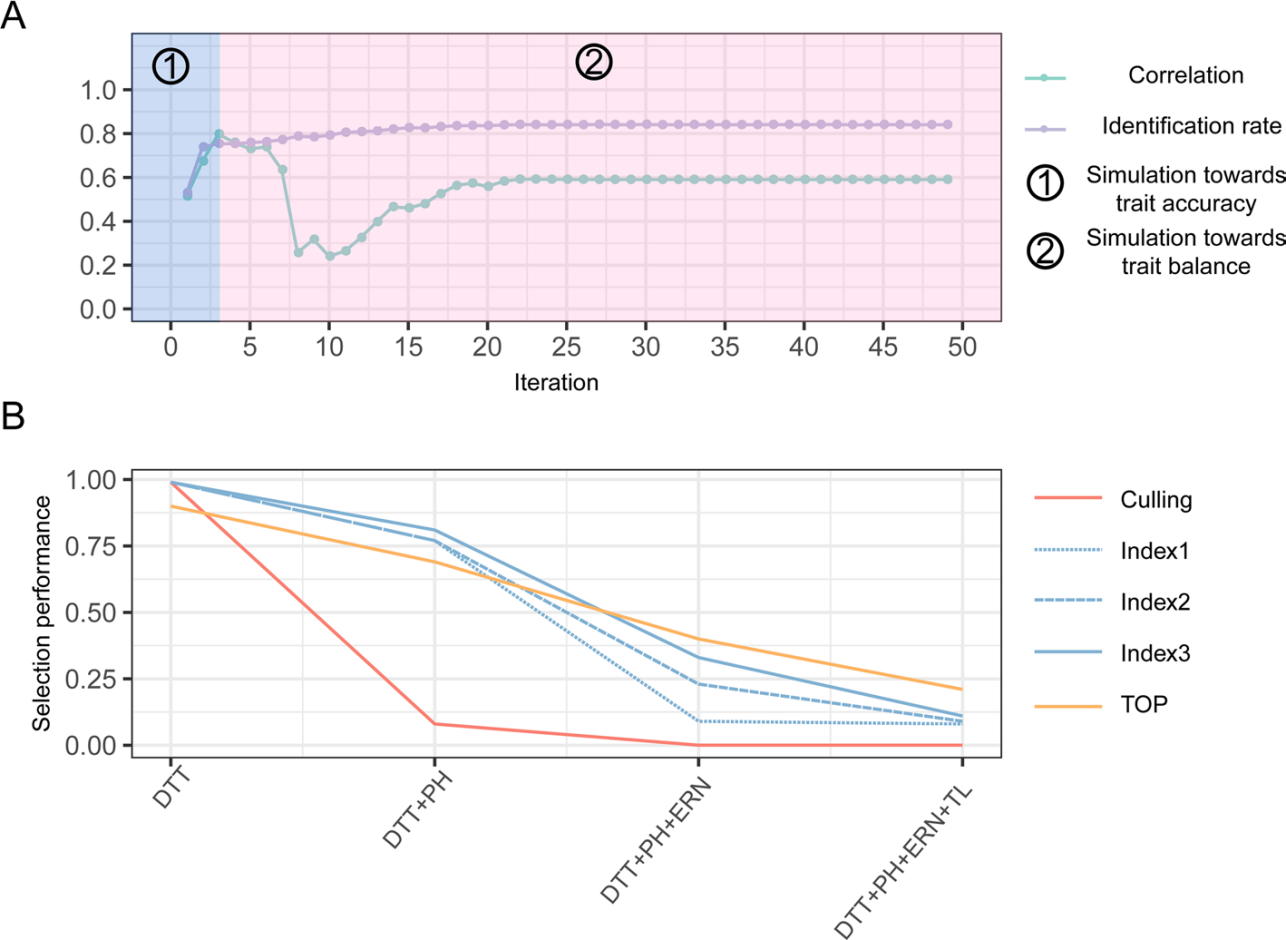
The performance of multi-trait selection methods. **A** The learning workflow of TOP algorithm. It iteratively learned the trait weights, the whole process firstly learned the weights by towards trait prediction accuracy (assessed by correlation between each round of simulated weight and prediction accuracy), then attempted to learning the trait balance status. The process converged until the identification rate goes stable. **B** The performance of multi-trait selection methods. Three methods are tested, including (i) independent culling levels (red line); (ii) three scenarios of weights on economic value for index selection called Index1, Index2, and Index3 (blue dash line, blue bold dash line, and blue line); and (iii) TOP (orange line)

We further explored the optimal weights learned by TOP in the multi-trait similarity analytic process. First, the uniformed weights, the initial values for weight settings in the TOP algorithm, for multi-trait similarity are apparently better than roughly selection by chance. The identification rate appeared to be raised by testing trait prediction accuracy as trait weights, while the optimal weights outputted by the TOP algorithm hit the best (Additional file 1: Fig. S4). To understand how the TOP algorithm work to learn the optimal trait weight, we recorded the temporal weights for each learning iteration and tested the proximity with trait accuracy assessed by correlation between each round of simulated weight and prediction accuracy. It was found that the part of early iterations may focus on learn the trait prediction accuracy very fast, the subsequent iterations may adjust the trait weights for further enhancing the identification accuracy. The learning process will converge until the identification rate became stable as the multi-trait synergistic status of weights was achieved (Fig. 3A). Furthermore, we tested the performance of the TOP algorithm with other selection schemes on multiple traits and found that the TOP algorithm outperformed the culling and index selection method when simultaneously considering more than two traits in realistic breeding scenarios (Fig. 3B).

### Identifying breeding candidates in maize association panels and rice bi-parental population

We tested the integrative multi-trait breeding strategy in three additional datasets to explore its versatility and reliability (see the “Methods” section). Two of the datasets were association panels, including 368 diverse inbred lines from China and 282 diverse inbred lines from the US, called Maize368 and Maize282, respectively. The other was a rice bi-parental population with 210 recombinant inbred lines (Rice210).

**Fig. 4:**
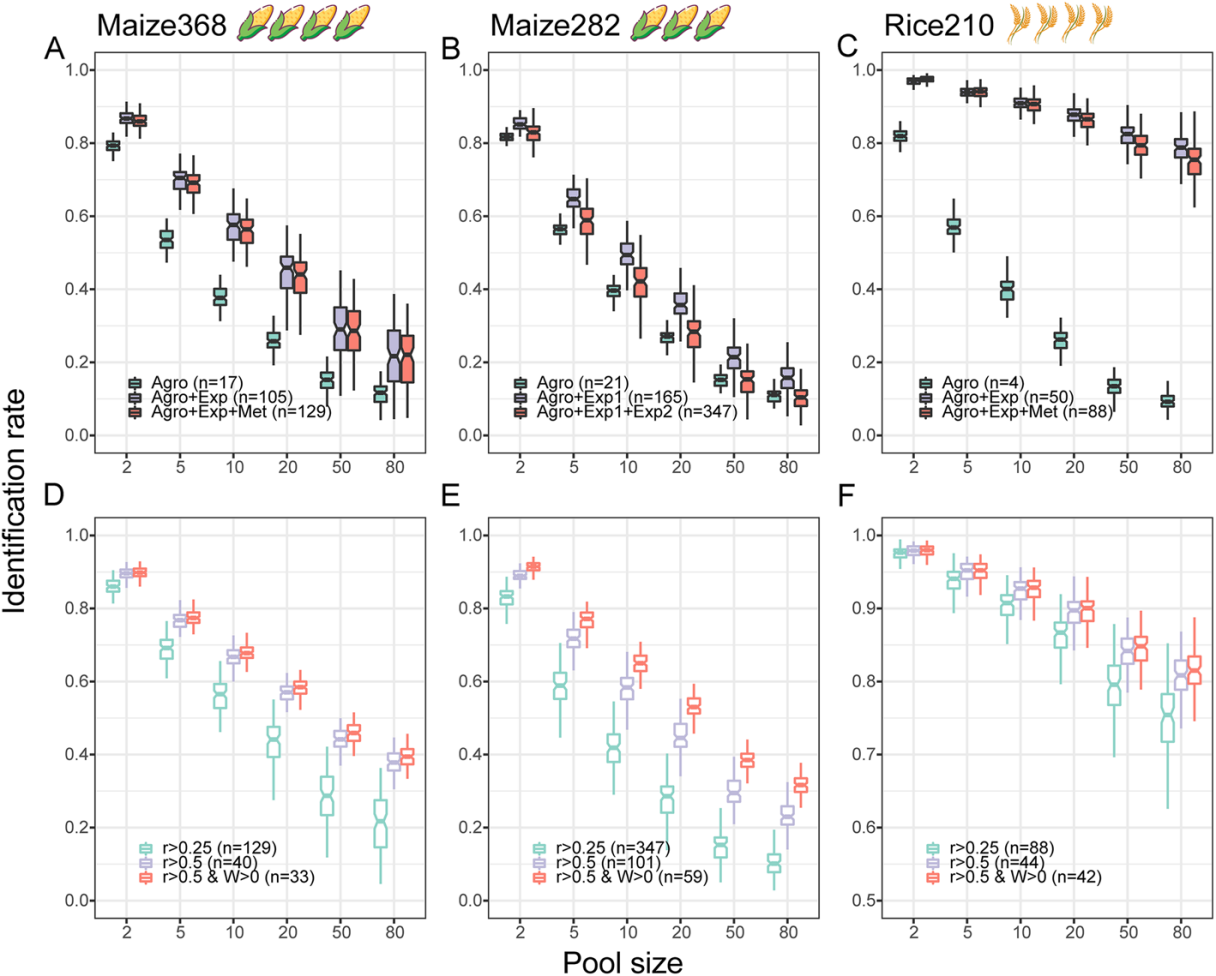
Improvement of TOP accuracy driven by robust omics data. **A**–**C** Identification rate of TOP increases when more omics traits are included in the model. For the Maize368 dataset, 17 agronomic traits (Agro), 88 transcriptomic traits (Exp), and 24 metabolic traits (Met) were sequentially added in the TOP model; For the Maize282 dataset, 21 agronomic traits, 144 transcriptomic traits from developing tissues (Exp1) and 182 transcriptomic traits from adult tissues (Exp2) were sequentially added in the TOP model; For Rice210 dataset, 4 agronomic traits (Agro), 46 transcriptomic traits (Exp), and 38 metabolic traits (Met) were included. All omics data with single-trait prediction accuracy less than 0.25 were excluded from the analyses. **D**–**F** Identification rate improvement due to filtering low-quality data. Before model training, traits with prediction accuracy (*r*) greater than 0.5 were considered; after training, traits with poor weights (*w*<0) were excluded from the model

Maize368 were genotyped with 1.25M SNPs and phenotyped for 17 agronomic traits, 88 transcriptomic traits, and 24 metabolic traits. To validate the ability of the machine learning algorithm to select superior breeding candidates in this dataset, we performed TOP with agronomic traits first, then adding transcriptomic traits, and then further adding metabolic traits. We found that TOP’s identification rate significantly increased after adding transcriptomic traits (*P*=1.35E−25; Fig. 4A) but did not change much when further adding metabolic traits. Maize282 was characterized with 50,878 SNPs, 21 agronomic traits, 144 transcriptomic traits from developing tissues, and 182 transcriptomic traits from adult tissues. A similar pattern was seen in Maize282 analyzed with TOP as was seen in Maize368, and the identification rate increased significantly from agronomic traits alone to agronomic plus transcriptomic traits together (*P*=2.66E−7; Fig. 4B). In the Rice210 dataset of 270,820 SNPs, 4 agronomic traits, 46 transcriptomic profiles, and 38 metabolites were analyzed with TOP. A high identification rate was seen when either of the one set of 46 transcriptomic traits or 84 combined traits were analyzed with 4 agronomic traits (*P*=3.36E−216; Fig. 4C). With the pool size increased, the identification rate was largely reduced in the two maize datasets, while it remained high (~0.8) in the Rice 210 even when the pool size reached 80 individuals. This result demonstrated that the selection efficiency of the algorithm can be boosted by adding molecular and cellular traits which are easily available by multiplex omics technologies, while the performance may be varied due to the population diversity.

It is worthy to note that simply adding more traits will not necessarily improve the TOP accuracy, as illustrated that adding metabolic traits appeared to reduce the identification rate from agronomic and transcriptomic traits (Fig. 4A, B). Motivated by the hypothesis that those traits less predictable may introduce noise rather than the helpful information for training the TOP algorithm, we tested the algorithm performance by excluding traits with low prediction accuracy (*r*). Indeed, by keeping predictable traits only (*r*>0.5), the identification rate in Maize368 significantly increased across all pool sizes (*P*=1.48E−20). In particular, a nearly 2-fold accuracy increase occurred in the largest pool of 80 individuals (Fig. 4D). An improvement was also observed in Maize282 (*P*=1.28E−20; Fig. 4E) and Rice210 (*P*=7.23E−12; Fig. 4F). A fraction of traits were exceedingly poor predictors and were estimated to have negative weights after running the learning process in TOP; and excluding these traits improved identification rates further, especially in Maize282 (Fig. 4D–F). This improvement may be due to the reduction of model complexity and measurement errors.

### Selecting hybrids that outperform an existing commercial variety

A segregating maize NCII population with 5251 F_1_ hybrids was used from which to select hybrids whose performance exceeds an existing model commercial variety. This model variety served as the ideotype that TOP was tested against to identify target(s) that are improved versions of certain traits. We chose Zhengdan958 as the model variety as it is an elite commercial maize hybrid and has been widely grown in the Chinese Corn Belt in the past decades [26]. Two versions of target hybrids were specified, one flowering 5% earlier than Zhengdan958 (early version) and one 5% later (late version). As a control, Zhengdan958 was itself included in the testing set as a target.

**Fig. 5:**
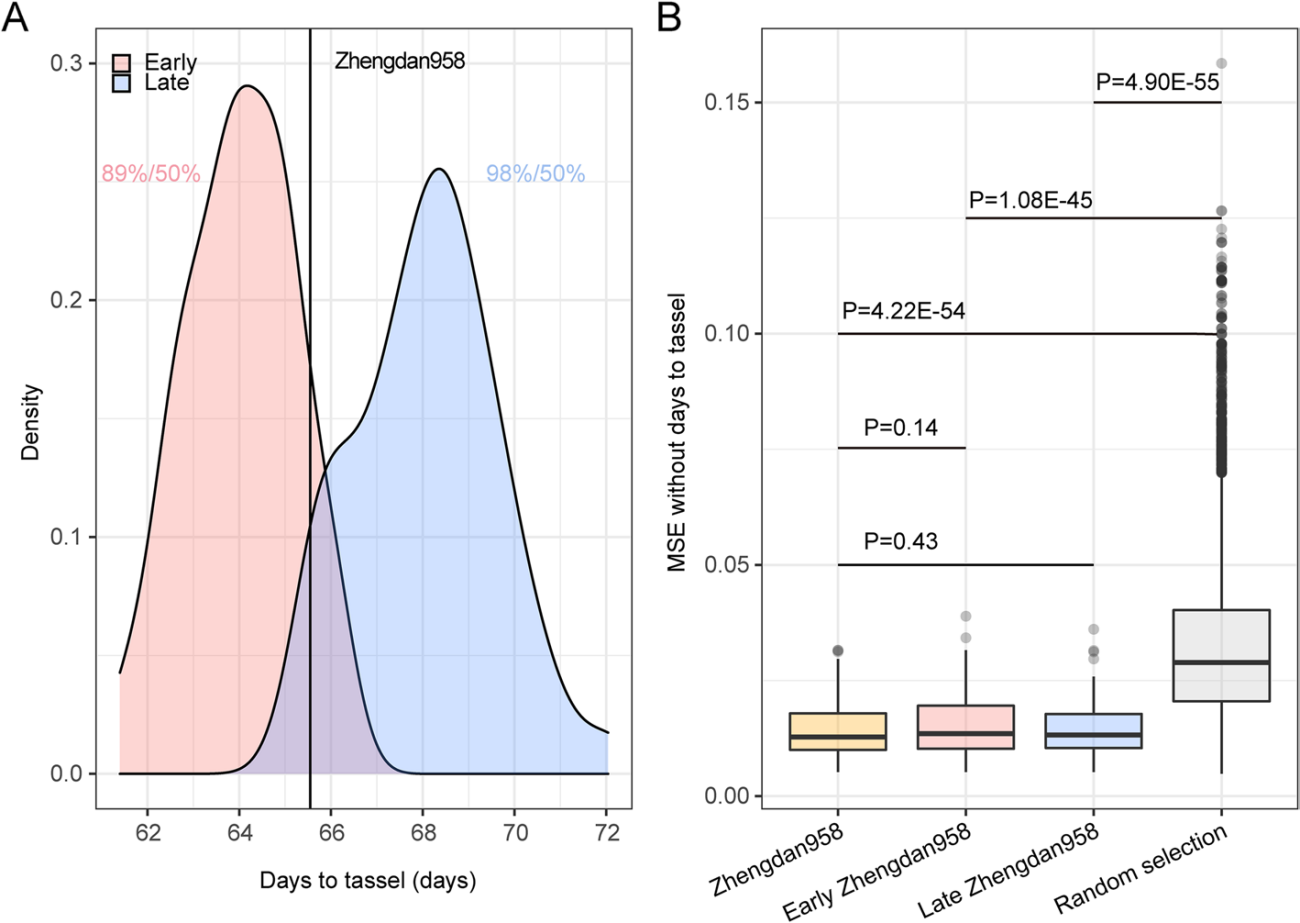
Selecting individuals with either earlier or later flowering than Zhengdan958. **A** The distribution of flowering time of the 100 individuals most similar to the target, with the 5% earlier (red) or later (blue) flowering individuals relative to Zhengdan958 (the black vertical line). The proportion of individuals selected with earlier and later flowering compared to Zhengdan958 is indicated by the value before the slash in red and blue, respectively, while the proportion of randomly selected individuals is after the slash in both cases. **B** The global similarity between selected individuals and Zhengdan958. The global similarity is measured by the mean squared error (MSE) for all traits excluding days to tassel between each selected individual and Zhengdan958; lower MSE values indicate higher global similarity. Three selection scenarios, early-version Zhengdan958 (red), late-version Zhengdan958 (blue), and Zhengdan958 itself (yellow), are presented for comparison with the randomly selected individuals, based on Student’s *t* test

TOP was run to identify the 100 hybrids most similar to the Zhengdan958 early version target in the Maize NCII population. Of these, 89 hybrids were found to actually flower earlier than Zhengdan958 (Fig. 5A). TOP was then run to identify the 100 hybrids most similar to the Zhengdan958 late version target, 98 of which flowered later than Zhengdan958. The proportion of TOP successful selection are significantly higher than random selection. Because the target improved upon the model variety only with respect to flowering time and maintain the characteristics of all other traits, we validated the expectation that the 100 selected target candidates remained similar to Zhengdan958 for all traits except flowering time. As a result, the hybrids traits’ resemblance to Zhengdan958 was much higher for candidates selected by TOP than those selected randomly (Fig. 5B). Similar encouraging results were obtained by TOP when identifying hybrids for improved plant height (PH) or ear weight (EW) (Additional file 1: Fig. S5 and S6). To illustrate how target position will influence TOP performance, we additionally chose four hybrids as targets located at the 10th, 20th, 80th, and 90th percentile of the distribution of flowering time in the maize NCII population. We found that the extreme target will reduce the successful selection rate in any approaches, but the extreme situation may further highlight the usefulness of our method in enrichment of elite candidate hybrids than random selection (Additional file 1: Fig. S7).

**Fig. 6:**
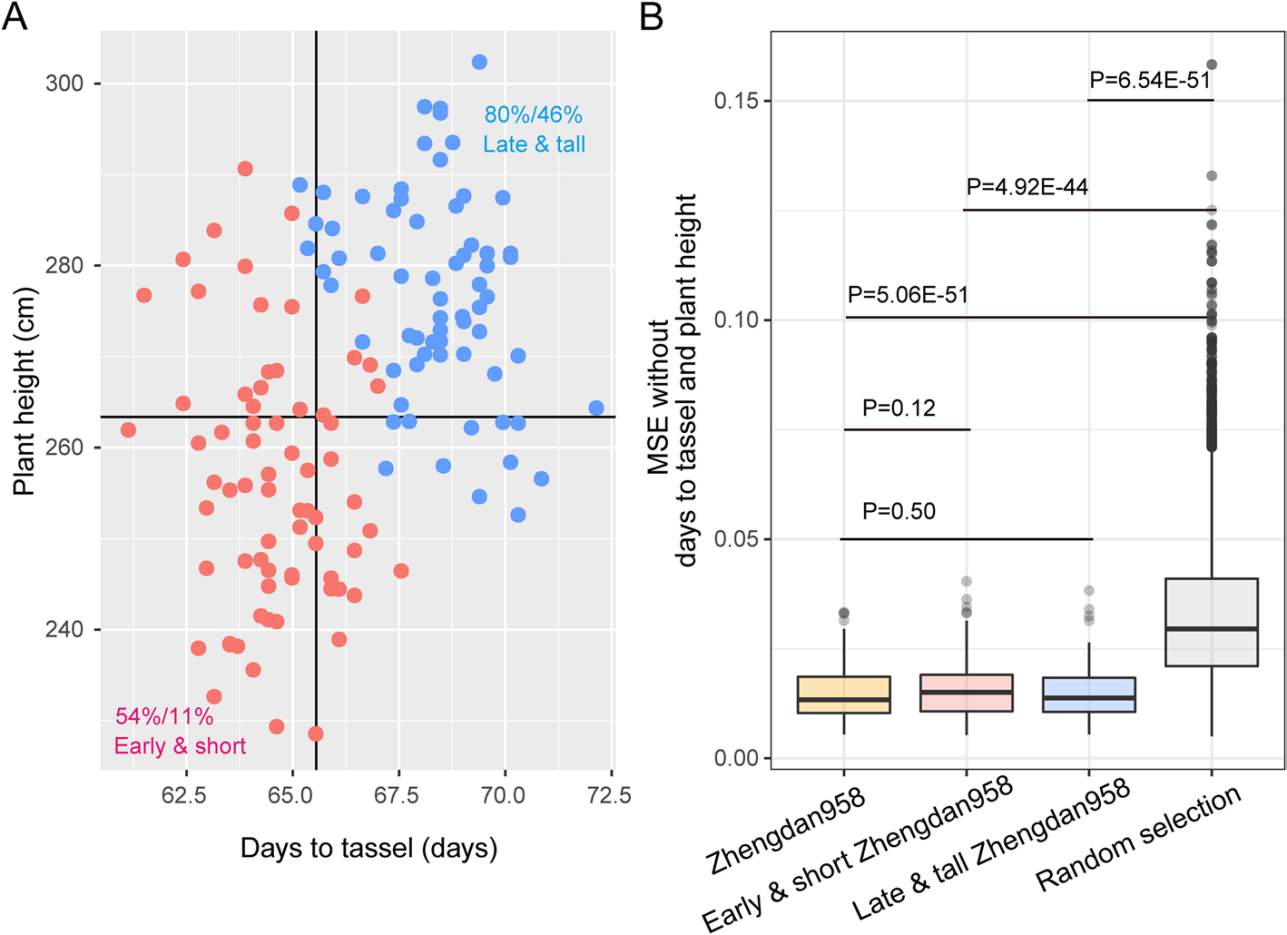
Selecting individuals with earlier flowering and shorter plant stature than Zhengdan958. **A** Scatter plot of flowering time and plant height of selected individuals. The red dots indicate the earlier flowering, shorter (early and short) version of Zhengdan958, and the blue dots indicate the late and tall version. The black vertical and horizontal lines indicate flowering time (days to tassel) and plant height (cm) of Zhengdan958. The proportion of individuals selected by TOP as early & short compared to Zhengdan958 is indicated by the percentage before the slash in red, and late & tall in blue, and the proportion of individuals selected randomly after the slash in both cases. **B.** The global similarity between selected individuals and Zhengdan958. The similarity measurement excluded days to tassel and plant height. Three selection scenarios, early and short, late and tall, and original version of Zhengdan958, were compared with randomly selected individuals based on Student’s *t* test

We then targeted improvement of Zhengdan958 for two traits simultaneously: flowering time and plant height. Two contrasting target versions were specified, one with a 5% decrease in both flowering time and plant height (early and short), and one with a 5% increase (late and high). As a control, Zhengdan958 was itself again included in the testing set as a target. We found that the hybrids selected by TOP were phenotypically highly similar to the target for multiple traits. More specifically, when hybrids were randomly selected, only 11% of them fell into the domain of early flowering and short plants and 46% into the domain of late flowering and tall plants. In contrast, TOP increased these percentages to 54% and 80%, respectively (Fig. 6A). In addition, the hybrids selected by TOP all resembled Zhengdan958 in respect to all traits except for the two manipulated traits (flowering time and plant height), much more so than the hybrids randomly selected (Fig. 6B). Hybrids that improve upon Zhengdan958 by manipulating different combinations of other traits are presented in Additional file 1: Fig. S8 and S9. Conclusively, TOP is effective for selecting candidate targets improved for specific trait characteristics while maintaining the desired aspects of an existing commercial variety or other ideotypes.

Early maturity, short plant height, and high yield are the crucial goal in maize breeding. We tried using our algorithm to assist design hybrid combinations. We previously systematically predicted three traits (flowering time, plant height, and ear weight) of 34,188 potential hybrids with 1221 maternal lines and 28 paternal lines that had not been actually created [27]. Based on the genomic prediction of three traits, the TOP algorithm recommended 86 F_1_ combinations from 15 maternal lines and 3 paternal lines that may predictively perform better than the commercial maize hybrid varieties (Zhengdan958, Xianyu335, and Jingke968) as check lines. We accordingly created the 86 hybrids and tested the field performance of three traits compared to check lines. The field data demonstrated over a quarter of predictive F_1_ had superior ear weight while kept relatively stable or even early flowering time and lower plant height compared to the check lines (Additional file 1: Fig. S10). We found that the F_1_ hybrids outperforming check lines were crossed from Jing89, DengHai351, and 5831, the elite inbred lines with the Reid\improved Reid pedigree. The check line “Zhengdan958” also had the Reid pedigree from its maternal part “Zheng58.” The 10 hybrids superior to Zhengdan958 had the significant ear weight improvement by 5.84% on average (0.75–8.66%), while maintaining relatively stable flowering time and plant height (within ±5%). The variety “Jingke968” has the characteristics of high ear weight and tall plant height. We still found four hybrids had superior performance than Jingke968 in simultaneously ear weight, flowering time, and plant height. These hybrid combinations showed the potential for maize yield breeding programs by balancing and simultaneously improving early maturity and logging resistance.

## Discussion

Genomic selection is a useful approach to predict trait performance in large populations [28]. This approach permits the breeder to select individuals with favorable values of traits in a cost- and labor-efficient manner, but generally focuses on a single or few key traits in each cycle of the selection process [11, 20]. However, breeders must be able to improve multiple traits simultaneously in order to create modern cultivars with attributes of high and stable yield, biotic and abiotic stress resistance, and multiple end-point uses for agricultural production. Selection for optimal expression of multiple traits is challenging due to the correlations among target traits. The use of a selection index allows the selection of individuals using a linear combination of predicted values of multiple traits [29], but the indices assigned to traits can be biased by personal experience and are population or environment dependent, such that the selection results may deviate from the expectation.

Building on the strength of machine learning, we present an integrative multi-trait breeding strategy that uses target-oriented prioritization (TOP) to first learn the similarity between genomic-predicted values and measured phenotypic values and then to predict the degree of similarity between inbreds or hybrids and the target with respect to hundreds of traits. This strategy resulted in high identification accuracy in a maize NCII population, two maize association panels, and one rice recombinant inbred line population, suggesting robustness and reliability of this strategy for identifying target candidates. TOP was also used to improve an existing commercial variety for key traits. The resulting hybrids selected by TOP not only retained the favorable characteristics of the commercial variety, but improved on unfavorable aspects. For instance, we found the 100 candidates selected for the early Zhengdan958 target significantly enriched two major haplotypes compared to random selection (*P*<0.01), and the selection of earlier flowering than Zhengdan958 is likely due to the higher frequency of the early allele of the *VGT1* gene (Additional file 1: Fig. S11). The current algorithm of the TOP method is to search the similarity of all traits to an improved target where one trait was modified while others were kept the same. In the future, we will test the performance by combining TOP and culling selection, where one trait was culled after TOP selection on other traits was done.

The explosive growth of omics data provides new techniques for efficient crop breeding. Improved genomic prediction models that allow the integration of omics data have proven effective in a rice study, in which inclusion of metabolic data almost doubled prediction accuracy over using genomic data alone [30]. Given the inherent hierarchical structure of omics data, a directed learning strategy provides an alternative approach to integrate multi-layer omics data for trait prediction. This approach predicted transcriptomic and metabolic layer data from genomic data and used them to predict phenotypic trait expression, a strategy that outperformed genomic prediction [31]. These methods enhance trait prediction by fitting more omics-based predictor variables into the statistical model. Compared with the single trait prediction method, recent progress on multi-trait prediction methods can improve further prediction accuracy [32, 33]. MegaLMM is a multi-trait genomic prediction based on the multivariate linear mixed effect model and can efficiently leverage thousands of traits at once to enhance prediction accuracy [33]. Moving beyond single-trait or multi-trait prediction methods, TOP learns the similarity between genomic predicted values and phenotypic values measured at levels of the whole organism, organs, tissues, cells, and macromolecules, and predicts the degree of similarity between untested genotypes and the target, which can be a preferred commercial variety. However, a significant amount of omics data may be biased if included when they are barely controlled by genetics, the cautious eyes and better algorithms for huge data may be required. As TOP is an integrative strategy that employs genomic prediction, omics, phenomics, and machine learning, improvement of these technologies [34–36] are also expected to boost TOP performance.

## Conclusions

Aiming to optimize the decision-making of selection of multiple and often nonsynergistic traits in breeding, we proposed a machine learning method for integrative multi-trait breeding strategy named TOP. The new method incorporates trait predictions at both whole-plant and molecular levels to make a cohesive decision for selecting superior candidate individuals by maximizing the overall similarity to an ideotype performance. We demonstrated the reliability and robustness of TOP by the real data from multiple populations and species and independent validation of field trial. In the era of surging biologically big data, the TOP method will provide efficient and valuable breeding decisions in the process of searching the large-scale germplasm resources for the high-yield and climate-resilient varieties.

## Methods

### Datasets

The maize NCII population includes 5820 F_1_ hybrids created from the cross of 194 maternal inbred lines which were a subset of the maize Complete-diallel plus Unbalanced Breeding-derived Inter-Cross (CUBIC) population [37] and 30 diverse elite paternal lines. We called 13.8 million single nucleotide polymorphisms (SNPs) in all 224 inbred lines by whole-genome resequencing and the pipeline was described in the publication of the CUBIC population [37]. We used PLINK [38] to filter out SNPs with minor allele frequency (MAF) <0.05 or expected missing rate >10% in the hybrid population, and to prune SNPs with an LD threshold of 0.3. We used the resulting 156,269 SNPs for the 5820 F_1_ hybrids for analysis. Missing genotypes in the hybrids due to heterozygosity of either parent was imputed using Beagle V4.0 [39].

The 5820 F_1_ hybrids were grown in five locations in 2015 for phenotype collection. A total of 18 agronomic traits were measured and include flowering traits (days to tassel, days to anther, and days to silk), plant architecture traits (plant height, ear height, ear leaf width, ear leaf length, tassel length, tassel branch number) and yield traits (cob weight, ear weight, ear diameter, ear length, ear row number, kernel number per ear, kernel number per row, kernel weight per ear, length of barren tip). The measurements of these traits followed standard procedures described previously [37]. The best linear unbiased predictor (BLUP) values were calculated and used as phenotypic data for further analysis.

The Maize368 dataset consists of 368 maize inbred lines from a widely used maize association mapping panel [40]. The 368 inbred lines were genotyped by multiple platforms and characterized with ~1.25M high-quality SNPs as reported by Liu et al. [41]. RNA sequencing, or RNA-Seq, was performed on RNA extracted from the immature kernel from each of the 368 lines 15 days after pollination (15DAP), yielding expression data from 28,768 genes [42]. An untargeted primary metabolomic profile detected 749 metabolite variables in the 368 inbred lines across three environments [24]. Seventeen agronomic traits from a previous GWAS analysis [43] were used in this study. The transcriptomic and metabolic variables were composed into 88 and 24 traits, respectively, via principal component analysis (PCA) based on the cumulative variance of 80%.

The Maize282 dataset consists of 282 maize inbred lines from a US maize association mapping panel [44]. All inbred lines were genotyped with 50,878 SNPs by Illumina MaizeSNP50 BeadChip [45] and phenotypically scored for a total of 21 agronomic traits including flowering time, plant architecture, yield, and disease resistance [44]. RNA-Seq was performed from RNA collected in each of the 282 inbred lines from seven tissues, including germinating root, germinating shoot, third leaf from the base, third leaf from the top, adult leaf collected during the day, adult leaf collected at night, and mature kernel [46]. In total, 144 transcriptomic traits (principal components) were obtained by composing variables from developing tissues and 182 transcriptomic traits were from adult tissues with a cumulative variance of 80%.

The Rice210 dataset is comprised of 210 recombinant inbred lines created from crossing between two rice varieties Zhenshan 97 and Minghui 63 [47]. A total of 1619 bins (no recombination exists within a bin) were identified from 270,820 SNPs by sequencing all the lines using next-generation sequencing [48]. The 1619 representative SNPs, one per bin, were used as the genomic data of the Rice210 dataset. A transcriptomic profile was created from RNA collected from the flag leaf using a microarray sequencing platform, quantifying the expression of 24,994 genes in total [49]. A metabolic dataset was collected, including 683 metabolites measured from the flag leaf and 317 metabolites from germinated seeds [50]. Four agronomic traits were available, including yield per plant, tiller number per plant, grain number per panicle and 1000 grain weight [51]. In total, 46 and 38 transcriptomic and metabolic traits (principal components) with a cumulative variance of 80% were obtained by composing variables from RNA-Seq and metabolomics data analysis, respectively.

### Genomic best linear unbiased prediction

The genomic best linear unbiased prediction (GBLUP) approach based on a mixed linear model was used for trait predictions. The formula is expressed as: *y* = *Xb* + *Zμ* + *ε*, with $\mu \sim N\left(0,K{\sigma}_g^2\right)$ and $\varepsilon \sim N\left(0,I{\sigma}_e^2\right)$, where *y* is an *n* × 1 vector of phenotypic values of a quantitative trait for *n* individuals; *X* is an *n* × *p* design matrix; *b* is a *p* × 1 vector of fixed effects, *Z* is an *n* × *n* design matrix; *μ* is an *n* × 1 vector of random effects representing individual genetic values with the variance-covariance matrix *K*, also known as genomic relationship matrix estimated by the software GEMMA [52]; *ε* is an *n* × 1 vector of residual errors; *I* is an identity matrix; and ${\sigma}_g^2$ and ${\sigma}_e^2$ are the estimated genetic variance and residual variance, respectively. The genetic values of all individuals were obtained with the following equation: *μ* = *KV*^−1^(*y* − *Xb*), where *b* = (*X*^*T*^*V*^−1^*X*)^−1^(*X*^*T*^*V*^−1^*y*), and $V=K{\sigma}_g^2+I{\sigma}_e^2$. The genomic prediction was implemented in the R package “rrBLUP” [53] and the Pearson’s correlation coefficient (*r*) between predicted and observed values was used to measure the prediction accuracy.

For testing the TOP algorithm, the whole population (5820 hybrids) was divided into training and testing set. The algorithm required predicted and measured trait values for the same individuals for learning model and testing performance. First, the training set of 569 hybrids was used train the GBLUP model for predicting the testing set. Second, to obtain the predicted trait values of the training set, we further divided 569 hybrids into 10 parts; for any one part of hybrids, the predicted values can be obtained by training the GBLUP model in the remaining 9 parts; ten rounds of iterations enabled to generate all predictions in the 569 hybrids.

### The target-oriented prioritization procedure

Target-oriented prioritization (TOP) is a flexible machine learning algorithm that integrates predictions of multiple traits for identifying a breeding candidate with maximized similarity to a target entry (hybrid maize in this study). TOP was implemented using the following steps: (1) in the training population, a similarity function was used to connect the predicted and measured traits for each individual; (2) through machine learning in the training population, the optimal weights of multiple traits were obtained, indicating the importance of individual traits in maximizing the similarity; and (3) in the testing population, the individuals that were the most similar phenotypically to a target were selected based on the degree of similarity, calculated with genomic-predicted phenotypes, observed phenotypes of the target entry, and optimal weights learned in the training population.

To validate the reliability of the TOP algorithm, we split the testing population into many pools, each comprised of *N*_0_ individuals, where *N*_0_ is from 20 to all individuals in the testing population. From the pool of *N*_*0*_ individuals, a randomly picked individual was defined to be the target (O_1_), the phenotypes across multiple traits of O_1_ were compared with a given individual (O_2_) from *N*_*0*_ individuals. If TOP identified O_2_ as a candidate based on the highest degree of similarity between genomic-predicted phenotypes with observed phenotypes of the O_1_ target, and the O_2_ is exactly the same to the O_1_, identification was successful. Because the target O_1_ was picked randomly from the pool of *N*_*0*_ individuals, we ran the identification procedure *N*_0_ times by considering each individual from the pool as an O_1_. The identification rate is the proportion of successful identifications of O_2_ (*N*_1_/*N*_0_, where *N*_1_ is the number of successful events) and was defined as the accuracy of TOP.

### The similarity function

A similarity function between multi-trait predicted and observed values was defined and learned in the training population. The similarity function was expressed as:

$$P\left({Y}^n,{\hat{Y}}^n\right)=\frac{\mathit{\exp}\left(-{\sum}_{i=1}^d{w}_i\left|{Y}_i^n-{\hat{Y}}_i^n\right|\right)}{\sum_{n=1}^N\mathit{\exp}\left(-{\sum}_{i=1}^d{w}_i\left|{Y}_i^n-{\hat{Y}}_i^n\right|\right)},$$

where ${Y}_i^n$and ${\hat{Y}}_i^n$ are observed and predicted values, respectively, of the trait *i* in the individual *n*, *d* is trait number, *N* is the training population size, and *w*_*i*_ is the weight for the trait *i*. For omics data, traits here refer to the principal components that retain most (>80%) of the variation presented in the original transcriptomic or metabolic features.

The optimal weights of multiple traits were obtained by maximizing the likelihood function $L(w)={\prod}_{n=1}^NP\left({Y}^n,{\hat{Y}}^n\right)$. This maximizes the following function: $\ln \left(L(w)\right)={\sum}_{n=1}^N lnP\left({Y}^n,{\hat{Y}}^n\right)$ with respect to the weights. The BFGS method published simultaneously in 1970 by Broyden, Fletcher, Goldfarb, and Shanno was used to solve the above optimization problem [54], with initial weights set to one.

In the testing population, the similarity degree was defined by the above similarity function in the training population, denoted by $P\left(Y,{\hat{Y}}^n\right)$,

where *Y* is the observed value of a given target; ${\hat{Y}}^n$ is the predicted value of the individual *n* in the testing population.

### The BFGS optimization algorithm

The BFGS method is one of the quasi-Newton methods that are well-known methods in solving unconstrained optimization problems. In general, the unconstrained optimization problems are described as follows: $\underset{x\in {R}^n}{\min }f(x)$, where *R*^*n*^ is an *n*-dimensional Euclidean space and *f* : *R*^*n*^ → *R*. The iterative formula for the quasi-Newton methods is defined as: *x*_*k* + 1_ = *x*_*k*_ + *α*_*k*_*d*_*k*_, ${d}_k=-{B}_k^{-1}{g}_k$, where *α*_*k*_ is the step size, *d*_*k*_ is the search direction, *g*_*k*_ is the gradient of *f* at *x*_*k*_, and *B*_*k*_ is an approximation of Hesssian of *f* at *x*_*k*_. The computation of BFGS algorithm is described as follows:

- Step 1. Given a starting point *x*_0_ and *B*_0_ = *I*_*n*_.
- Step 2. Terminate if ‖*g*_*k*_‖<10^−6^.
- Step 3. Calculate $${d}_k=-{B}_k^{-1}{g}_k$$.
- Step 4. Calculate *α*_*k*_ by a line search.
- Step 5. Compute *s*_*k*_ = *x*_*k* + 1_ − *x*_*k*_ and *y*_*k*_ = *g*_*k* + 1_ − *g*_*k*_.
- Step 6. Calculate *x*_*k* + 1_ = *x*_*k*_ + *α*_*k*_*d*_*k*_, ${B}_{k+1}={B}_k-\frac{B_k{s}_k{s}_k^T{B}_k}{s_k^T{B}_k{s}_k}+\frac{y_k{y}_k^T}{s_k^T{y}_k}$, and go to Step 2.

In this paper, let − ln(*L*(*w*)) be the above function *f*, then the optimal weight *w* can be obtained using the BFGS method.

### A comparison of three multi-trait selection methods

In the method of “independent culling levels” a genotype is culled if it does not meet the requirement for a single trait, regardless of its levels on other traits. Considering one trait, independent culling levels was run to select 100 superiority hybrids from testing population. When multiple traits are considered, the above process is repeated several times, and the selected hybrids are intersected.

Index selection assigns weights to different traits relative to their economic importance. The index is a linear combination of phenotypic trait values defined as: *I* = *b*^*T*^*X*, where *X* is *n* × 1 phenotypic vector and *b* is an *n* × 1 weight vector calculated as:

$$b={p}^{-1}{G}^{\ast }{\left[{G^{\ast}}^T{p}^{-1}{G}^{\ast}\right]}^{-1}Q$$

where *Q* is an *m* × 1 (*m* < *n*) vector of desired genetic gains, *G*^∗^ is an *n* × *m* matrix which is derived from additive genetic variance-covariance matrix *G* by keeping the *m* columns for the traits that appeared in *Q*. Then economic values can be calculated by *a* = *G*^−1^*pb*.

In this paper, we set three scenarios for *Q*: i) a 5 % reduction in DTT (Days to tassel), a 5 % reduction in PH (Plant height), a 5 % increase in ERN (Ear row number), and a 5 % increase in TL (Tassel length); ii) a 5 % reduction in DTT, a 5 % reduction in PH, a 10 % increase in ERN, and a 5 % increase in TL; iii) a 10 % reduction in DTT, a 15 % reduction in PH, a 20 % increase in ERN, and a 5 % increase in TL. Hence, three scenarios of weights on economic value obtained corresponding to Index1, Index2, and Index3.

Take the ERN trait for example, if the trait value of a select hybrid is 0.5 times the standard deviation larger than the ERN mean value of the testing population, the selection is considered a success for the above two methods. For the TOP method, if the difference between the trait value of a select hybrid and the corresponding trait value of the target less than 0.5 times the standard deviation, the selection is considered a success. The selection performance of the three methods is defined as the number of successes divided by the selection number denoted by NS (NS=100).

### Field trial for 86 F_1_ combinations designed by TOP algorithm

We previously systematically predicted three traits (flowering time, plant height, and ear weight) of 34,188 potential hybrids with 1221 maternal lines and 28 paternal lines, using the GBLUP model trained in 8632 hybrids [27]. Based on the predicted and measured values of three traits, a TOP algorithm was trained in 8,632 hybrids. We deployed three commercial hybrid varieties (Zhengdan958, Xianyu335, and Jingke968) as the control, and the improved versions of three controls as the targets, with a 10% decrease in flowering time and plant height while a 20% increase in ear weight for the controls. We searched 34,188 hybrid combinations by TOP algorithm, while considering the parental pedigree and ecological adaptation in low-latitude region, ultimately determined 86 hybrid combinations had the high probability of better trait performance than the controls. We then established the 86 hybrids manually in the experimental station of Beijing in 2019. The 86 hybrids were grown in the Hainan experimental station in the winter of 2019 for plot-based phenotyping. We used an augmented design which included systematic checks (Zhengdan958, Xianyu335, and Jingke968) multiple times, which are usually used in the maize breeding experiments. Each hybrid or check was grown in a three-row plot with 15 plants per row, 20 cm between plants, and 30 cm between rows. Each of the check plots was iteratively set per 9 hybrids. The flowering time and plant height were investigated in the middle row for each plot to reduce the marginal effects. The plot-based ear weight was measured by averaging over 30 normal developed ears for each hybrid and check.

## Supplementary Information

**Additional file 1.** Figures S1-S11.

**Additional file 2.** Target-Oriented Prioritization (TOP) tutorial.

**Additional file 3.** Review history.

## Data Availability

The demo R scripts and tutorial of the TOP algorithm can be publicly accessed at the GitHub [55], which is licensed under the GNU General Public License v3.0. The source codes are also available publicly at the Zenodo [56]. The genotypic and phenotypic data of the maize NCII population, Maize368, Maize282, and Rice210 populations were publicly accessible in the database figshare [57].
