# Supplementary material for: Target-oriented prioritization: targeted selection strategy by integrating organismal and molecular traits through predictive analytics in breeding
Source: Genome Biol. 2022 Mar 15;23:80. doi: 10.1186/s13059-022-02650-w (PMC8922918; doi:10.1186/s13059-022-02650-w)
Supplement: Supplementary file 1 — Additional file 1. Figures S1-S11. [file 13059_2022_2650_MOESM1_ESM.docx]

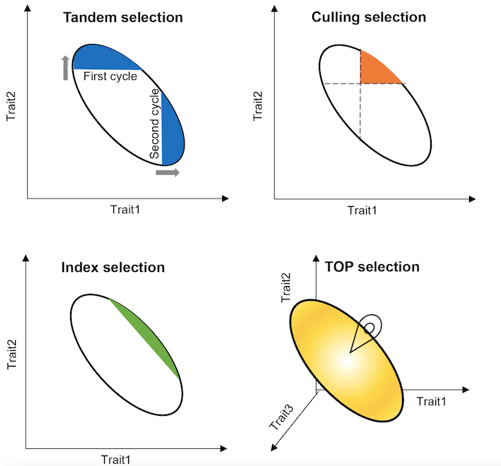


**Figure S1** Four selection schemes in breeding, including tandem selection, culling selection, index selection, and target-oriented prioritization (TOP) algorithm. Graph axes indicate expected gain from selection intensity applied to each trait.


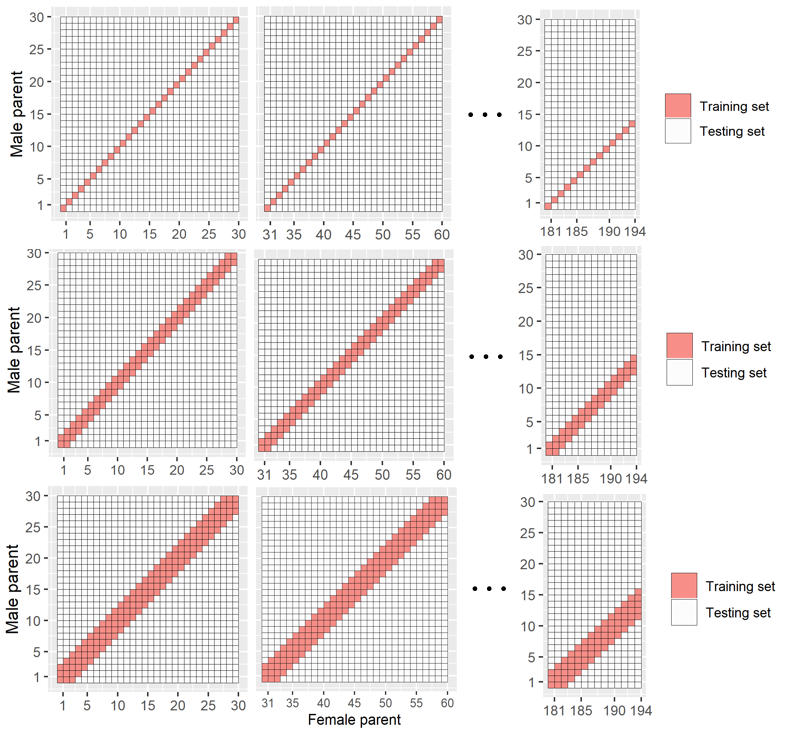


**Figure S2** The partition of training and testing sets for genomic prediction in the maize NCII population. The maize NCII population is composed of 5810 F_1_s with 194 female and 30 male lines. To select the optimal training set, we tested five partition schemes by selecting F_1_s from 1 to 5 diagonal strips as training set (red boxes), and the remaining F_1_s as testing set (white boxes). The top, middle and bottom panel shows the 1 diagonal, 3 diagonal and 5 diagonal strips, respectively.


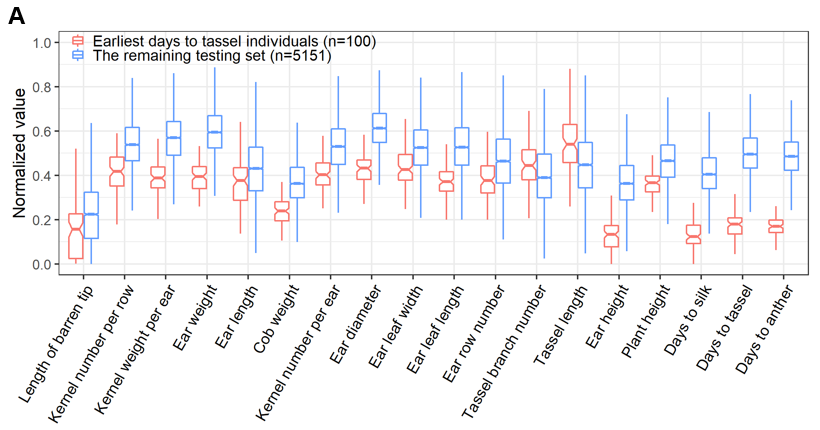


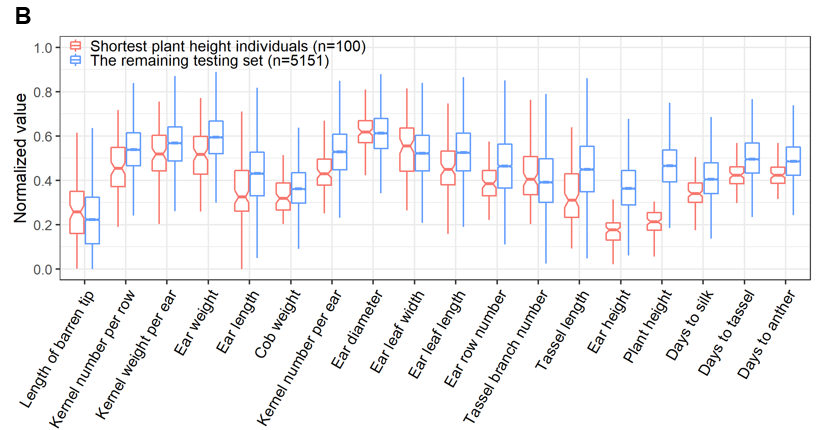


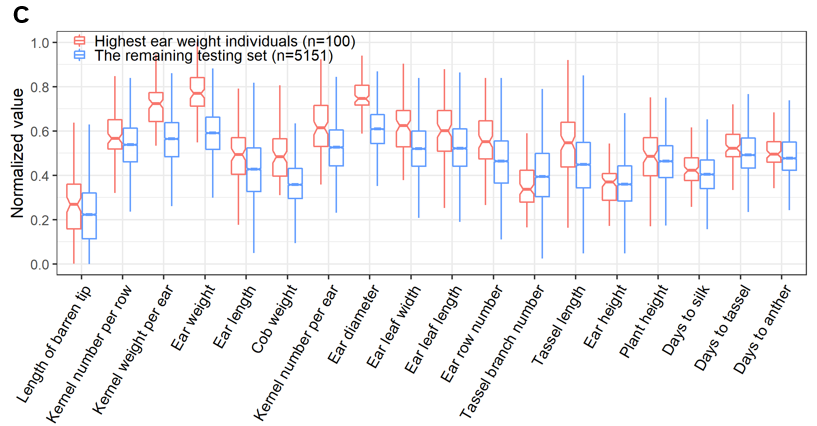


**Figure S3** The selection performance based on trait predictions. **A**) Selection for early flowering. **B**) Selection for short plant. **C**) Selection for increased ear weight. For each trait, the 100 most extreme individuals were selected (red) for comparison with the remaining individuals (blue) from the 5251 F_1_ hybrids, across all traits, based on the Student’s t test (P<0.01).


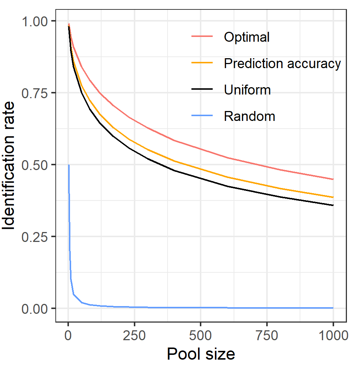


**Figure S4** The relationship between identification rate and model parameter setups. Four parameter setups are tested, including: i) uniformed weights, i.e., 1s for all traits (black line); ii) prediction accuracy as weights without model learning (orange line); iii) optimal weights learned by the TOP model (red line); and randomly selected (blue line).


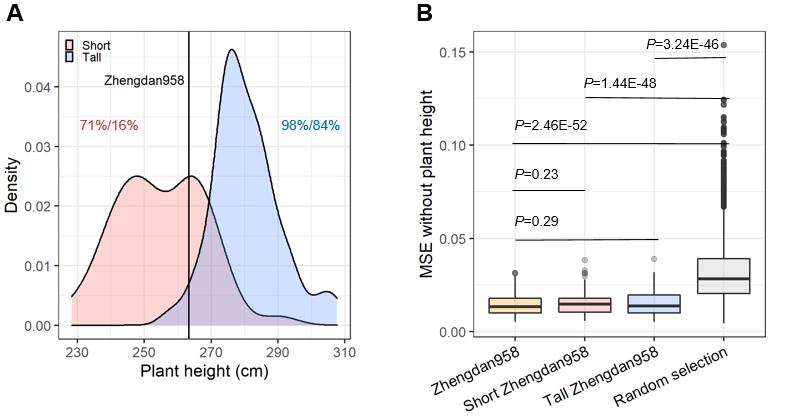


**Figure S5** Selecting an improved, shorter version of Zhengdan958. **A**) The distribution of plant height of the 100 individuals most similar to the target, with the 5% shorter (red) or taller (blue) plants compared to Zhengdan958 (the black vertical line). The proportion of individuals selected by TOP with shorter and taller plants compared to Zhengdan958 is indicated by the percentage before the slash, and the proportion of individuals selected randomly after the slash. **B**) The global similarity between selected individuals and Zhengdan958. The global similarity is measured by the mean squared error (MSE) for all traits excluding plant height between each selected individual and Zhengdan958; lower MSE value indicates higher global similarity. Three selection scenarios, short-version Zhengdan958 (red), tall-version Zhengdan958 (blue) and Zhengdan958 (yellow), are presented for comparison with the randomly selected individuals, based on Student’s t test.


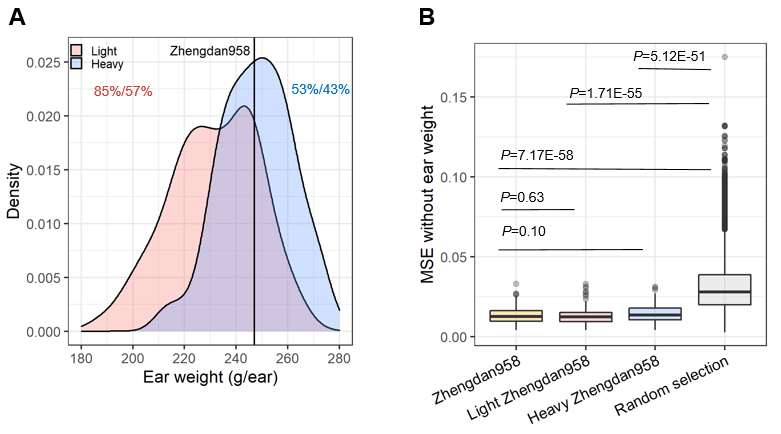


**Figure S6** Selecting an improved, higher yielding version of Zhengdan958. **A**) The distribution of ear weight of the 100 individuals most similar to the target, with the 5% lighter (red) and heavier (blue) ears compared to Zhengdan958 (the black vertical line). The proportion of individuals selected by TOP with smaller and larger ears compared to Zhengdan958 is indicated by the percentage before the slash, and the proportion of individuals selected randomly after the slash. **B**) The global similarity between selected individuals and Zhengdan958. The global similarity is measured by the mean squared error (MSE) for all traits excluding ear weight between each selected individual and Zhengdan958; lower MSE value indicates higher global similarity. Three selection scenarios, light-version Zhengdan958 (red), heavy-version Zhengdan958 (blue) and Zhengdan958 (yellow), are presented for comparison with the randomly selected individuals, based on Student’s t test.


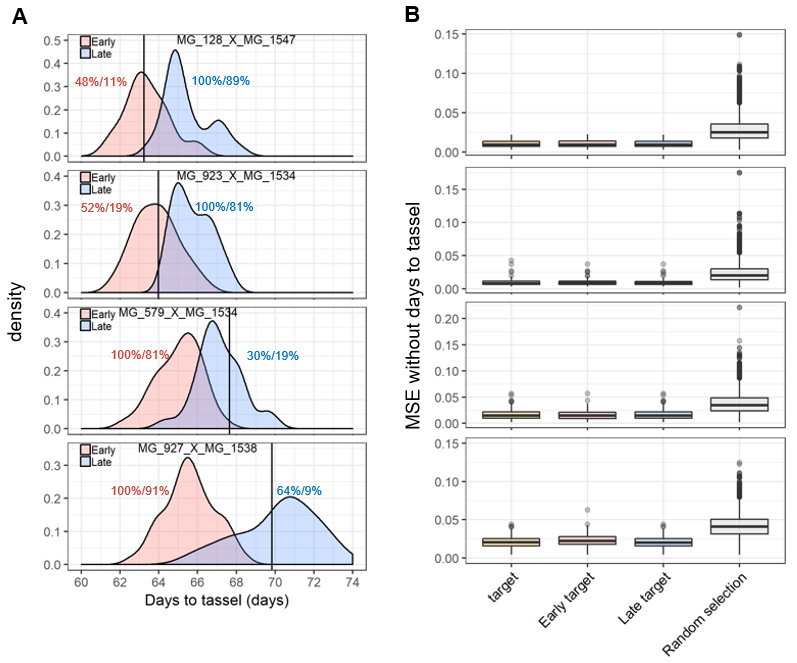


**Figure S7** Selection performance of early version of targets with different distribution locations. In the maize NCII population, four hybrids with flowering time at the 10^th^ (MG_128_X_MG_1547), 20^th^ (MG_923_X_MG_1534), 80^th^ (MG_579_X_MG_1534) and 90^th^ (MG_927_X_MG_1538) percentiles of the distribution. A) The distribution of flowering time of the 100 individuals most similar to the target, with the 5% earlier (red) or later (blue) flowering individuals relative to the target (the black vertical line). The proportion of individuals selected with earlier and later flowering compared to the target is indicated by the value before the slash in red and blue, respectively, while the proportion of randomly selected individuals is after the slash in both cases. B) The global similarity between selected individuals and the target. The global similarity is measured by the mean squared error (MSE) for all traits excluding days to tassel between each selected individual and the target; lower MSE values indicate higher global similarity. Three selection scenarios, early-version target (red), late-version target (blue) and the target itself (yellow), are presented for comparison with the randomly selected individuals, based on Student’s t test.


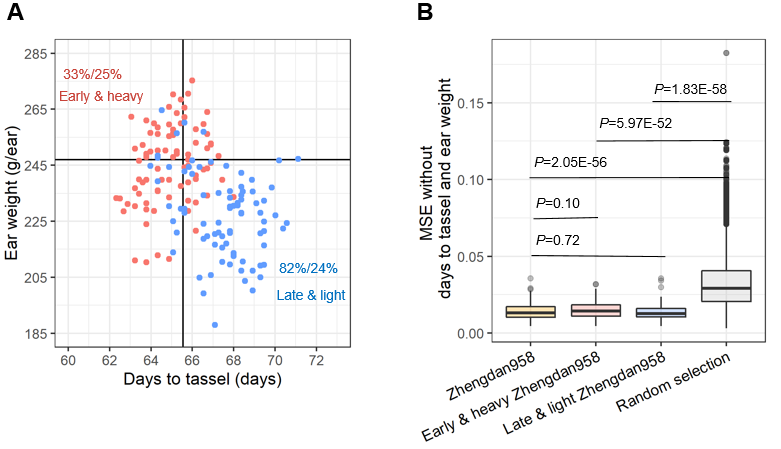


**Figure S8** Selected an improved, earlier flowering and higher yielding version of Zhengdan958. **A**) Scatter plot of flowering time and ear weight of the 100 selected individuals. The red dots indicate the earlier flowering, heavier ear (early & heavy) version of Zhengdan958, and the blue indicates the late & light version. The black vertical and horizontal lines indicate flowering time (days to tassel) and ear weight (grams/ear) of Zhengdan958. The proportion of individuals selected by TOP as early & heavy compared to Zhengdan958 is indicated by the percentage before the slash in red, and late & light in blue, and the proportion of individuals selected randomly after the slash in both cases. **B**) The global similarity between selected individuals and Zhengdan958. The similarity measurement excluded the traits days to tassel and ear weight. Three selection scenarios, early & heavy, late & light and the original version of Zhengdan958, were compared with randomly selected individuals based on Student’s t test.


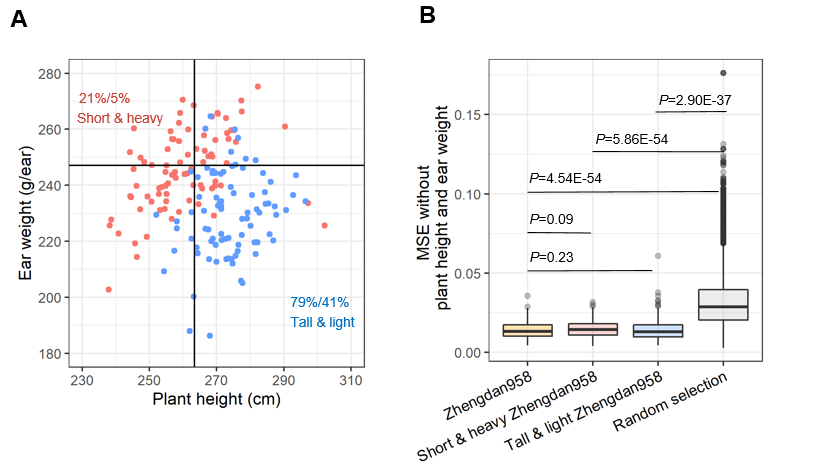


**Figure S9** Selected an improved, shorter and higher yielding version of Zhengdan958. **A**) Scatter plot of plant height and ear weight of the 100 selected individuals. The red dots indicate shorter, heavier ear (short & heavy) version of Zhengdan958, and the blue indicates the tall & light version. The black vertical and horizontal lines indicate the plant height (cm) and ear weight (grams/ear) of Zhengdan958. The proportion of individuals selected by TOP with shorter plants and heavier ears compared to Zhengdan958 is indicated by the percentage before the slash in red, taller plants with lighter ears in blue, and the proportion of individuals selected randomly after the slash in both cases. **B**) The global similarity between selected individuals and Zhengdan958. The similarity measurement excluded the traits days to tassel and ear weight. Three selection scenarios, short & heavy, tall & light and the original version of Zhengdan958 were compared with randomly selected individuals based on Student’s t test.


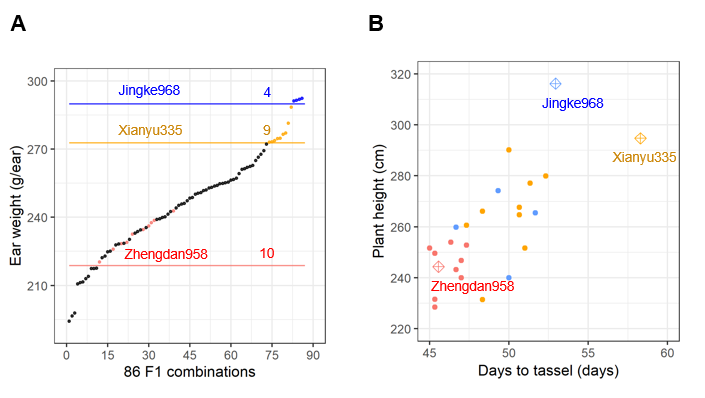


**Figure S10** The result of the field trial of the 86 combinations selected by TOP. A) Ear weight of 23 hybrids superior to check lines in independent trial. There were 10, 9 and 4 F_1_ hybrids were considered to be superior to three check lines (Zhengdan958, Xianyu335 and Jingke968), respectively, that the flowering time and plant height were relatively stable or even less than one of check lines, while ear weight was greater. B) The flowering time and plant height of the F_1_ hybrids superior to three check lines.


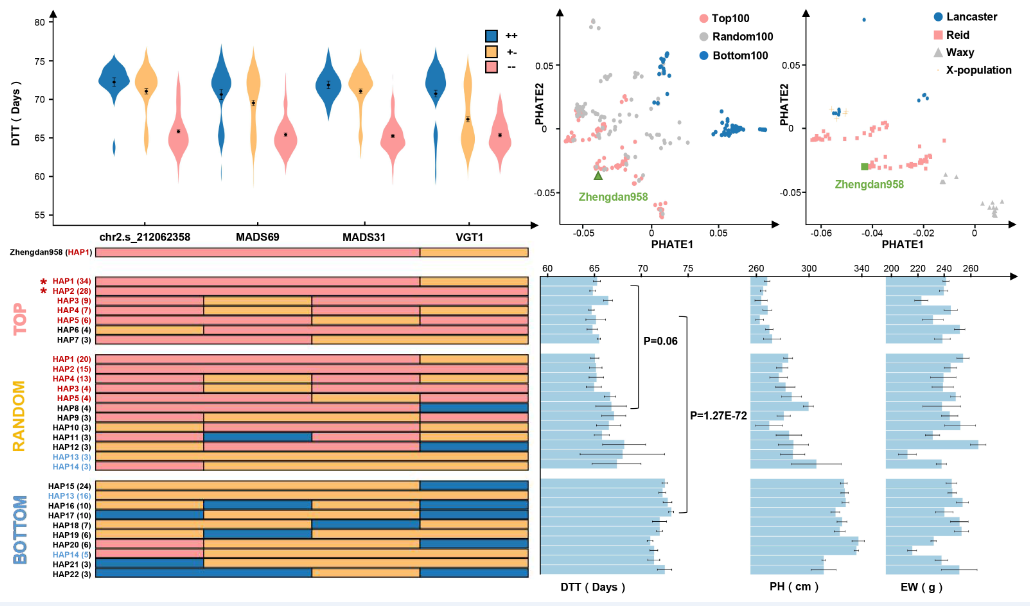


**Figure S11** Genetic basis of selecting candidate hybrids for early Zhengdan958. Four major QTLs that were reported in GWAS for days to tasseling in the previous CUBIC publication (Liu et al., 2020, Genome Biology) were used in this analysis. The early allele of four genes or loci were indicated by red color (**top-left panel**). The candidates with the top 100, random 100 and bottom 100 similarity scores were distinctly clustered, and the top 100 candidates predominantly originated from the Reid heterotic group (**top-right panel**). The top 100 candidates were significantly enriched for two haplotypes (marked by asterisk) compared to random selection (P<0.01). The Zhengdan958 haplotype is improved by substituting an early allele at *VGT1* locus (**bottom panel**).
