# Supplementary material for: Target-oriented prioritization: targeted selection strategy by integrating organismal and molecular traits through predictive analytics in breeding
Source: Genome Biol. 2022 Mar 15;23:80. doi: 10.1186/s13059-022-02650-w (PMC8922918; doi:10.1186/s13059-022-02650-w)
Supplement: Supplementary file 2 — Additional file 2. Target-Oriented Prioritization (TOP) tutorial. [file 13059_2022_2650_MOESM2_ESM.docx]

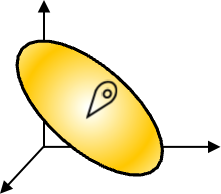


**TOP - T**arget-**O**riented **P**rioritization

Version 1.0

Updated at 2022.01.23

Wenyu Yang ([yangwenyurain@126.com](mailto:yangwenyurain@126.com))

Jianbing Yan ([yjianbing@mail.hzau.edu.cn](mailto:yjianbing@mail.hzau.edu.cn))

Yingjie Xiao ([yxiao25@mail.hzau.edu.cn](mailto:yxiao25@mail.hzau.edu.cn))

**1 Introduction**

Genomic selection (GS) is a useful approach to predict trait performance in large populations. This approach permits the breeder to select individuals effectively. Upon GS, the decision-making of selection for multiple end-point use in agriculture, high yield, good quality and environmental resilient, is challenging due to the nonsynergistic relations among traits. Target-Oriented Prioritization (TOP) is a machine learning algorithm, provides an integrative multi-trait breeding strategy that incorporates trait predictions at both whole-plant and molecular levels to make a cohesive decision for selecting superior candidates (Fig. 1).


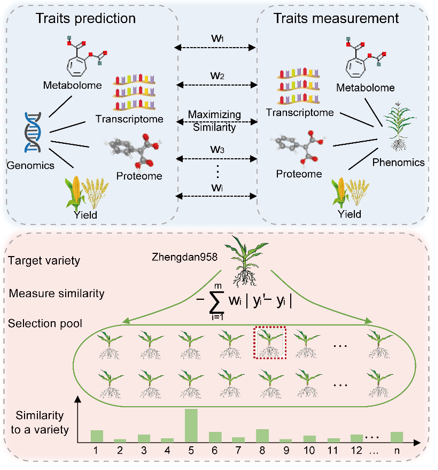


**Fig. 1 Multiple selection schemes in crop breeding.** The schematic workflow of the TOP algorithm. By learning the optimal trait weights using the maximum likelihood algorithm, genomic predictions of multiple traits are integrated to select the best individual candidates from diverse breeding pools, maximizing the global similarity to an ideotype or target.

Compared to traditional breeding approach, our TOP method has two advantages: i) this algorithm proposes a brand-new genomic breeding angle, shifting from a traditional way that select the superior subset individuals based on phenotype, to the direct identification of individuals via projecting multiple genomic predictions to an ideotype performance; ii) this method can mathematically learn the synergistic or competing relationships among multiple traits, which help to wisely make decisions for multi-end selections. iii) the method has the potential for selecting candidates that outperform an existing commercial hybrid variety.

The usage of TOP script include:

1. Trait prediction from genomic data using popular algorithm (e.g., GBLUP).
2. Identifying trait weights for selection using machine learning.
3. Select candidate hybrids that most assemble to the commercial cultivar.
4. Select elite hybrids superior to an existing commercial cultivar or check line.

**2 GETTING STARTED**

TOP method is compatible with R, which is user-friendly and easily distributed with free licenses. The analysis of TOP method requires an independent ‘rrBLUP’ package (Endelman 2011) for genomic prediction. But it’s noted that, the genomic prediction method is not limited to be rrBLUP, any state-of-art methods can be used.

Reference:

Endelman JB: **Ridge regression and other kernels for genomic selection with R package**

**rrBLUP**. *Plant Genome* 2011, **4**(3):250-255.

**3 INPUT**

**3.1 Genotypic file**

The genotypic file requires a header row, which shows the names of individuals. The first column shows the names of molecular markers or SNPs. For each SNP, the genotypes, e.g., AA, AT, TT, are required to transformed to integer format as 0, 1 or 2 based on allelic frequency. For example, A is the major allele that AA and TT will be transformed to 0 and 2, the AT is transformed to 1 (Table 1). It means the copy number of minor alleles for a line at a SNP locus, relative to major allele.

**Table 1 Genotypic format of 5 SNPs in 5 individuals**

| SNP | R001 | R002 | R003 | R004 | R005 |
| --- | --- | --- | --- | --- | --- |
| SNP1 | 0 | 0 | 2 | 0 | 0 |
| SNP2 | 0 | 0 | 0 | 0 | 2 |
| SNP3 | 0 | 0 | 0 | 0 | 0 |
| SNP4 | 0 | 0 | 0 | 1 | 0 |
| SNP5 | 2 | 2 | 2 | 2 | 2 |

**3.2 Agronomic trait file**

The agronomic trait file must contain a header row, which represents the names of traits. The first column shows the names of the individuals (Table 2). The trait values are numeric and missing values are indicated as NA, following the R coding requirement.

**Table 2 Agronomic format of 5 traits in 5 individuals**

| Ind | Trait1 | Trait2 | Trait3 | Trait4 | Trait5 |
| --- | --- | --- | --- | --- | --- |
| R001 | 80.41 | 79.52 | 1.58 | 166.04 | 73.17 |
| R002 | 82.35 | 81.44 | 1.41 | 169.06 | 92.27 |
| R003 | 71.24 | 70.17 | 1.46 | 161.59 | 78.06 |
| R004 | 82.18 | 79.81 | 2.36 | 156.90 | 83.03 |
| R005 | 65.66 | 65.53 | 0.68 | 120.44 | 41.33 |

**3.3 Omics trait file (optional)**

Our method can handle hundreds or thousands of molecular and cellular traits (omics data) for boost selection accuracy. Here, we provide the format for molecular traits using metabolic traits as an example (Table 3). As long data format is very easy to be processed and manipulated in R platform, the required data format treats the column as individuals and rows as metabolites. The header line is needed to identify the names of individuals, the first column indicates the names of metabolites. As mentioned above, the missing value is indicated as NA.

**Table 3 Omics format of 5 metabolites in 5 individuals**

| Meta | R001 | R002 | R003 | R004 | R005 |
| --- | --- | --- | --- | --- | --- |
| m0001 | 20.55729 | 20.66623 | 20.45463 | 20.77765 | 20.77013 |
| m0002 | 23.84497 | 23.71574 | 23.69052 | 24.10140 | 24.02722 |
| m0003 | 21.69904 | 21.78545 | 21.42181 | 22.28423 | 22.43024 |
| m0004 | 22.88953 | 22.65108 | 22.70262 | 23.18386 | 23.42256 |
| m0005 | 18.21402 | 18.13013 | 17.96329 | 18.40632 | 17.82904 |

|  |
| --- |
| \|  \| \| --- \| |

**4 USAGE**

**4.1 Before analysis**

To run TOP, the user requires to prepare three data files: genotypic file, agronomic data file, and omics data file (optional). This manual uses the metabolic data as an example of omics data. The user requires to keep all files in the same order of individuals before analysis.

**4.2 Read files**

The user could use following codes and demo data we provided to test the function of reading data from disk to R working space. It’s noted that, for novice users, the full path of data files that located in the computer is recommended to avoid possible errors.

***# read genotype***

Genotype <- read.csv("./data/demo_geno.csv",

header=TRUE,

sep=",",

stringsAsFactors=0,

check.names=FALSE

)

***# read agronomic trait***

Agro <- read.table("./data/demo_ agro.txt",

header=TRUE,

sep="",

stringsAsFactors=0,

check.names=FALSE

)

***# read metabolites***

Metabolite<-read.table("./data/demo_metabolites.csv",

header=TRUE,

sep=",",

stringsAsFactors=0,

check.names=FALSE

)

**4.3 Divide a population into training and test set**

To execute following analyses, the whole population requires to be divided into training and test sets. The user requires to prepare a text table for set information of individuals. It can be a two-column table, where the first column is the names of individuals (must be the same to the data files) and the second column is the set identity as training and test. The user could read the set information using following codes.

id <- read.table("./data/demo_training_test_id.txt",

header=TRUE,

sep="",

stringsAsFactors=0,

check.names=FALSE

)

**4.4 Obtain predicted values of all traits**

Based on the popular GBLUP model, implemented in the ‘*rrBLUP*’ package, we first obtain the trait predictions from genomic data. In our TOP method, we need to obtain predicted values in both training and test individuals. In the training set, trait predictions are for the TOP model learning. We use the 10-folds cross validation strategy to obtain predicted values, which predict 1 part from remaining 9 parts of training individuals and the process iterated 10 times. In the test set, trait predictions are for assessing the TOP accuracy. We use all training data to predict the individuals from test set.

We develop a user-friendly function ‘*Prediction_trait*’ for the analysis of this step. The tutorial code can be tested as followed:

Pre<-Prediction_trait(Genotype,

Agro,

Metabolite,

id,

pca=TRUE,

CEVR=0.8

)

*Notes:*

1. *Genotype: genotypic data of the population.*
2. *Agro: agronomic data of the population.*
3. *Metabolite: metabolic data of the population.*
4. *id: the names of individuals in the training and the test set.*
5. *pca: if the parameter “pca” is default set to be TRUE, PCA is performed to reduce the dimension of metabolic data.*
6. *CEVR: cumulative explained variance ratio of principal components. if the pca=TRUE, the default CEVR threshold is 0.8, means the subset of first principal components with CEVR>0.8 will be kept as metabolic traits for prediction.*

The trait predictions for training and test set can be extracted as followed:

prediction_train <- Pre$prediction_train

prediction_test <- Pre$prediction_test

**4.6 Obtain optimal weights of traits by learning TOP model**

In the training set, a machine learning algorithm is built to grid search a group of optimal weight values that represent the trait importance for accurately matching genomic predictions and measured phenotypes at the same individuals. If *m* traits are used to learn the TOP model, the function ‘*Weight_res*’ will output a numeric optimal weight vector of *mx1*. The user could test the tutorial codes as followed:

Optimal_Weight <- Weight_res(prediction_train,

trait_train,

names_trait,

b=0.25

)

*Notes:*

1. *prediction_train: the predicted trait values of the training set and the last row stores the average prediction accuracy of ten round.*
2. *trait_train: the observed trait values of the training set.*
3. *names_trait: the names of the traits.*
4. *b: the prediction accuracy threshold of entering the TOP model, the default is 0.25.*

**4.7 Test accuracy of TOP model**

Using the optimal weights learned from TOP model in the training set, the user can test the accuracy of matching genomic predictions and measured phenotypes in the independent test set. Technically, from a pool of individuals in test set (e.g., N=5), the trait predictions of all 5 individuals are tested the global similarities to their trait observations. If one individual’s predictions most match the trait observations itself, this is regarded to be successful identification. The proportion of success identification in a pool is defined to be the identification rate and TOP accuracy.

The user can use the function ‘*Test_top_acc*’ to estimate the accuracy of TOP method in current data.

TOP_acc <- Test_top_acc(prediction_test,

trait_test,

Weight=Optimal_Weight,

names_trait,

N=c(2,5,10),

m=200

)

*Notes:*

1. *prediction_test: the predicted trait values of the test set.*
2. *trait_test: the observed trait values of the test set.*
3. *Weight: indicate the importance of individual traits for searching global similarity to a target individual, the default is the optimal weight calculated by the TOP model.*
4. *names_trait: the names of the traits.*
5. *N: pool size for calculating TOP accuracy, the default is c(2,5,10).*
6. *m: the number of random permutations when selecting a pool of individuals from the test set, the default is 200.*

**4.8 Select elite individuals superior to an existing target**

To assist breeding, it is necessary to propose a list of elite individuals that has the superior performance in one or few primary trait (i.e., early maturity, disease resistance and high yield). Our method provides a manageable solution that selecting individuals with simultaneously superior trait(s) and relatively stable remaining traits. This approach may be helpful to bring improvement gains on selecting hybrids outperforming the widespread commercial cultivars. We provide a cohesive function ‘*Top_target*’ that integrats genomic predictions and optimal weights for searching elite individuals from the test set.

The user can test this function by running the following codes:

Top_target_sel <- Top_target(target,

prediction_test,

names_test,

names_trait,

pre_train_mean,

pre_train_sd,

obs_train_mean,

obs_train_sd,

selection_ratio=0.1,

improve_ratio=0.05,

improve_trait=4,

Weight=Optimal_Weight,)

*Notes:*

1. *target: a vector (1xd, d is the trait number) containing the values of each trait of a given target, often set as the trait observations of an existing commercial cultivar.*
2. *prediction_test: the predicted trait values of the test set.*
3. *names_test: the names of individuals in test set*
4. *names_trait: the names of the traits*
5. *pre_train_mean: the mean of predicted trait values of training set.*
6. *pre_train_sd: the standard deviation of predicted trait values of training set.*
7. *obs_train_mean: the mean of observed trait values of training set.*
8. *obs_train_sd: the standard deviation of observed trait values of training set.*
9. *selection_ratio: identify ratio of individuals in the test set with maximized similarity to the target, the default is 0.1, it indicates the top 10% similarity individuals will be selected.*
10. *improve_ratio: change ratio of a trait value from the target, the default is 5%, it indicates the improved target at this trait expect to have the phenotype 5% higher than the original one.*
11. *improve_trait: the trait ID of the target to be improved, if the ID is “NA”, no trait value of target will be modified; if the ID is “4”, the fourth trait in the list of all used traits will be modified.*
12. *Weight: indicate the importance of individual traits for searching elite candidates, the default is the optimal weights learned from TOP model.*

**5 OUTPUT**

**5.1 Output of trait weights learned by TOP model**

The function ‘*Weight_res*’ can learn the optimal weight of traits that maximizing the matching between genomic predictions and trait observations at the same individuals. The user can use *Optimal_Weight$W_matrix* to get the optimal weights which is a data frame including two columns: the first column are names of the traits of entering TOP model and the second column are optimal weights of the corresponding traits; The user can use *Optimal_Weight$* *pre_mean, Optimal_Weight$* *pre_sd, Optimal_Weight$* *obs_mean, Optimal_Weight$* *obs_sd* to get the mean of predicted trait values, the standard deviation of predicted trait values, the mean of observed trait values, and the standard deviation of observed trait values in the training test, which will be used in the function ‘*Top_target*’.

**5.2 Output of TOP accuracy**

The function ‘*Test_top_acc*’ calculates the TOP accuracy based on different pool sizes. The resulting *TOP_acc* variable contains two lists including identification rate of TOP model and an example of similarity matrix.

The user can use *TOP_acc$Ide_rate* to get the TOP accuracy at the pool size of 2, 5 and 10; The user can use *TOP_acc$Demo_p* to get the similarity matrix at the pool of 5 individuals (Fig. 2).


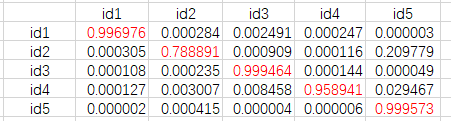


**Fig. 2 Similarity matrix of the pool size of 5 individuals.** The trait predictions of all 5 individuals are tested the global similarities to their trait observations. If one individual’s predictions mostly match the trait observations itself, this is regarded to be successful identification. The red number highlights the highest similarity with the target at each column.

**5.3 Output of selecting elite individuals superior to a target line**

The function ‘*Top_target*’ can search the breeding pool for select elite individuals that has one or few primary traits superior to target lines while relatively stable for the remaining traits. The resulting *Top_target_sel* variable returns two lists including the names of selected individuals and the predicted traits of selected individuals.

The user can use *Top_target_sel$names_select* to get the names of selected individuals that is a *1x10* vector in the demo; The user can use *Top_target_sel$valeus_select* to get the predicted values that is a *10x42* numeric matrix with 42 traits.

**5.4 Visualization of selection performance with field data**

If the traits observations of selected elite individuals are available by independent field trial, the user can visualize the performance of TOP selection relative to random selection. For example, if the fourth trait is expected to be improved 5% higher than the target line, the individuals with top 10% similarity and five rounds of random individuals with same size are selected. The trait observations of these selected by TOP method and random selection are compared for the improved trait#4 and the global similarity of remaining traits.

The user can use the function ‘*plot_TOP*’ to visualize the selection performance. This function requires the support of *ggplot2* package. The function *plot_TOP* returns two graphs: 1) density plot showing the trait#4 difference between individuals selected by TOP method and random selection; 2) the global similarity of remaining traits between the target and the individuals selected by TOP and random selection (Fig. 3).

Top_target_sel_name<-Top_target_sel$names_select

library(ggplot2)

plot_TOP (target,

trait_test,

Top_target_sel_name,

improve_trait=4,

group_random=5

)

*Notes:*

1. *target: a vector (1*d, d is the trait number) containing the values of each trait of a given target*
2. *trait_test: the observed trait values of the test set*
3. *Top_target_sel_name: the name of individuals selected by TOP*
4. *improve_trait: the trait to be improved*
5. *group_random: the number of groups of individuals randomly selected (the number of individuals in each group is the same as the number of individuals selected by TOP)*


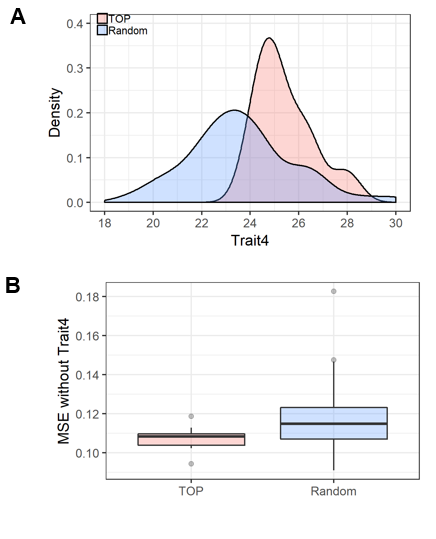


**Fig. 3 Selection performance of TOP method and random selection**
