# Supplementary material for: Target-oriented prioritization: targeted selection strategy by integrating organismal and molecular traits through predictive analytics in breeding
Source: Genome Biol. 2022 Mar 15;23:80. doi: 10.1186/s13059-022-02650-w (PMC8922918; doi:10.1186/s13059-022-02650-w)
Supplement: Supplementary file 3 — Additional file 3. Review history. [file 13059_2022_2650_MOESM3_ESM.docx]

Review History

**First round of review**

**Reviewer 1**

**Were you able to assess all statistics in the manuscript, including the appropriateness of statistical tests used?**

No, I do not feel adequately qualified to assess the statistics.

**Comments to author:**

The authors used the genetic data of CUBIC hybrid population and developed a new genomic prediction method. The design is generally nice. I'm not an expert on machine learning algorithm, so I cannot comment on the details of the algorithm. I will comment on the genetic section and the utility of the work.

1. The key validation of the algorithm is the development of new hybrid combinations for breeding. However, only one sentence (Line 254-256) and one supplementary figure (Figure S10) are present in the manuscript. This is obviously not enough. The details of several hybrids exceeding the performance of ZD958, XY335 and JK968 should be provided and analyzed in depth. The ear yield should be evaluated in multiple locations, with strictly designed plot experiments.

2. The conclusion is not suitable. It seems to be the conclusion of a review paper, not for an original research paper.

3. Any links between the genomic prediction method and known QTL genes in maize? The efforts in such links may provide new insights for maize community.

4. As a method/algorithm paper, benchmarking experiments are preferred for authors. Other software for genomic prediction can be tested using the same dataset (the CUBIC hybrids and the data of hybrid rice) and the performance of TOP can be compared with the others.

5. A step by step protocol of TOP in analyzing the dataset should be provided in Supplementary Text.

**Reviewer 2**

**Were you able to assess all statistics in the manuscript, including the appropriateness of statistical tests used?**

Yes, and I have assessed the statistics in my report.

**Comments to author:**

The article "Targeted selection by integrating organismal and molecular traits through predictive analytics" by Yang et al introduces and demonstrates a new multi-trait ML indexing approach for assessing and predicting the "similarity" of breeding lines against a commercial check, or ideotype.

They demonstrate that their tool is able to increase the proportion of elite candidates within the selection thresholds for target traits, while not depleting the value of other traits.

The authors provide all code and demo examples on GITHUB, which run on R version 4.1.0 (2021-05-18) Platform: x86_64-apple-darwin17.0 (64-bit) Running under: macOS 12.1.

This paper is well written and easy to understand because the objective was clear from the beginning, which is appreciated.

If I understand correctly (please correct me if I am wrong), the strategy is to use GBLUP for t traits (1 trait at a time) and then to make selections based on the 'superiority' of target trait t_1 among hybrids that have a high predicted similarity among target traits t_2,...,t_i. So in a way this is independent culling where the first cull trait is a selection index built from second/third priority traits?

I do have mostly minor comments and questions.

1. In general, the approach taken is to target similarity to some existing hybrid, e.g., Zhengdan958, Xianyu335, and Jingke968. I think this is creative, and allows researchers to take advantage of existing -omics data. However, I do not know how these three lines (especially Zhengdan958) compare to the rest of the population in terms of target traits. Figs 5 and 6, lead me to believe that Zhengdan958 must be mediocre in with respect to days to tassel and plant height. I understand that this hybrid is a popular cultivar, but I wonder if this example over emphasizes the performance of TOP? As an example, do you know what happens when yield is the target trait and the commercial check is in the upper tail of the distribution?

Specifically, I am curious how TOP performs when my ideotype is extreme for the primary target trait (ear weight). Perhaps this is what is being shown in Fig S6, S7, S8, and S10. However, Zhengdan958 "line" in S6, S7, S8 vid S10 seems to be in different places. from the first three, I think Zhengdan958 has g/ear = ~248, but in S10 is it plotted at 215 g/ear? maybe I am reading this wrong.

2. How would I use this in the case where my 'ideotype' is unobserved in my population?

3. Figure 2 only has A and B labels, but caption has A-D.

4. I feel that the multi trait GS references are a bit out of date and makes it seem that there has been limited improvement on multi-trait prediction, when in reality this is a rapidly growing space. I was able to find several more recent references with a quick google scholar search, e.g., Runcie et al 2021 - MegaLMM and Montesinos-López et al 2021 - multi-year wheat breeding trials. These approaches are different that yours, but are still part of this space.

5. "the similarity function"

Line 414-416 are redundant with 404-406 and I think there are missing super/sub scripts in 414-416.

"O" = observed and "Y" = predicted is non-standard and I would suggest using "Y" = observed and "Y_hat" = predicted.

I do not think that "N" or "d" are defined in this section? I believe that they are population size and trait number, respectively. Please make sure that all variables are defined as they appear in text.

6. How stochastic is the similarity matrix, i.e., similarity_matrix, in 'TOP_demo.r'? I ran the demo multiple times and got different results each time despite no changes to the code or the test/train set, etc. I suppose the stochasticity is from the individuals in the pools? I think that this needs more explanation in the code documentation.

> (similarity_matrix=ide_rate$Demo_p)

[,1] [,2] [,3] [,4] [,5]

[1,] 0.47245376 0.10275327 0.01271944 0.34940391 0.06266962

[2,] 0.04889945 0.33186839 0.03028727 0.04481262 0.54413227

[3,] 0.08440274 0.02529848 0.69900723 0.13965996 0.05163159

[4,] 0.12692922 0.03706160 0.01440462 0.74838112 0.07322343

[5,] 0.05898381 0.35695537 0.03075991 0.03849533 0.51480558

> (similarity_matrix=ide_rate$Demo_p)

[,1] [,2] [,3] [,4] [,5]

[1,] 0.55464817 0.125228438 0.187359011 0.12249032 0.010274055

[2,] 0.01311712 0.753341013 0.206353781 0.02692470 0.000263382

[3,] 0.03136113 0.543135510 0.399058223 0.02532194 0.001123195

[4,] 0.30236444 0.033900282 0.052966046 0.55701789 0.053751344

[5,] 0.06667338 0.002611329 0.009516418 0.04602473 0.875174149

Also, I am confused as to how I would use `Test_top_res()` to specify a specific target, e.g., Zhengdan958?

I am not entirely sure how to interpret the demo results and I think it would be beneficial to increase the the amount of documentation so that a complete novice could work with and interpret the code you have compiled.

It might also be helpful to build a demo where there is 1 specific target ideotype selected from the dataset.

7. "The transcriptomic and metabolic variables were composed into 88 and 24 traits, respectively, via principal component analysis (PCA)." What lead to this decision? Scree plot? variance explained? What is "the primary variation of the original transcriptomic or metabolic features."?

**Authors Response**

**Point-by-point responses to the reviewers’ comments:**

Reviewer 1#:

The authors used the genetic data of CUBIC hybrid population and developed a new genomic prediction method. The design is generally nice. I'm not an expert on machine learning algorithm, so I cannot comment on the details of the algorithm. I will comment on the genetic section and the utility of the work.

*Our response: Thank you for the nice comments.*

Comment 1: The key validation of the algorithm is the development of new hybrid combinations for breeding. However, only one sentence (Line 254-256) and one supplementary figure (Figure S10) are present in the manuscript. This is obviously not enough. The details of several hybrids exceeding the performance of ZD958, XY335 and JK968 should be provided and analyzed in depth. The ear yield should be evaluated in multiple locations, with strictly designed plot experiments.

*Our response: Thank you for the reviewer’s constructive suggestion. We have added an additional figure S10b that showed the performance of flowering time and plant height for the selected hybrids. We found that the F1 hybrids outperforming check lines were crossed from Jing89, DengHai351 and 5831, the elite inbred lines with the Reid\improved Reid pedigree. The check line “Zhengdan958” also had the Reid pedigree from its maternal part “Zheng58”. The 10 hybrids superior to Zhengdan958 had the significant ear weight improvement by 5.84% in average (0.75-8.66%), while maintaining the relative stable flowering time and plant height (within ±5%). The variety “Jingke968” has the characteristics of high ear weight and tall plant height. We still found four hybrids had the superior performance than Jingke968 in simultaneously ear weight, flowering time and plant height. These hybrid combinations showed the potential for maize yield breeding programs by balancing and simultaneously improving related early maturity and logging resistance. As the suggested by the reviewer, we had provided more statements for this section in the revised manuscript.*

*We agree that the ear yield need to be evaluated in multiple locations. Actually, in the research, we did perform the field trials in Henan province (the central province of China) in the years of 2020 and 2021. The bad weather of strong wind and storm happened in the maize growing seasons failed the whole field experiments, no valuable phenotypic data can be collected. The field trial in Hainan province in 2019 winter was strictly designed with data quality controlled. The 86 hybrids were grown in the Hainan experimental station. We used an augmented design which included systematic checks (Zhengdan958, Xianyu335 and Jingke968) multiple times, which are usually used in the maize breeding experiments. Each hybrid or check was grown in a three-row plot with 15 plants per row, 20 cm between plants and 30 cm between rows. Each of the check plots of were iteratively set per 9 hybrids. The flowering time and plant height were investigated in the middle row for each plot to reduce the marginal effects. The plot-based ear weight was measured by averaging over 30 normal developed ears for each hybrid and check. We added this detail of experimental design in the revised manuscript. The field trial in Hainan 2019 had given us the confidence for the beneficial breeding potential of 10 superior hybrid combinations, although impacted by bad weather and pandemic, we will continue the field evaluations in multiple locations and years in the future. We hope the reviewer can understand our situations in these unavoidable natural influences.*

Comment 2: The conclusion is not suitable. It seems to be the conclusion of a review paper, not for an original research paper.

*Our response: Thank you. We had rewritten the conclusion in the revised manuscript as “Aiming to optimize the decision-making of selection of multiple and often nonsynergistic traits in breeding, we proposed a machine learning method for integrative multi-trait breeding strategy named TOP. The new method incorporates trait predictions at both whole-plant and molecular levels to make a cohesive decision for selecting superior candidate individuals by maximizing the overall similarity to an ideotype performance. We demonstrated the reliability and robustness of TOP by the real data from multiple populations and species, and independent validation of field trials. In the era of surging biologically big data, the TOP method will provide efficient and valuable breeding decisions in the process of searching the large-scale germplasm resources for the high-yield and climate resilient varieties.”*

Comment 3: Any links between the genomic prediction method and known QTL genes in maize? The efforts in such links may provide new insights for maize community.

*Our response: We agree with the reviewer. In our research, we used the TOP method to select top 100 candidates for early version of Zhengdan958. Compared to by chance, the top 100 hybrids significantly enriched two major haplotypes (HAP1 and HAP2) based on a major QTL (chr2.s_212062358) and three known genes (MADS69, MADS31 and VGT1) for maize flowering time. From the analysis, we found the selection of early version of Zhengdan958 is probably due to the HAP1 enrichment, that caused by the higher frequency of the early allele of VGT1 gene (Figure S11). We believe the progress of functional genomics will further improve and interpret the genomic prediction of complex traits.*

Comment 4: As a method/algorithm paper, benchmarking experiments are preferred for authors. Other software for genomic prediction can be tested using the same dataset (the CUBIC hybrids and the data of hybrid rice) and the performance of TOP can be compared with the others.

*Our response: Thank you for the comments. Actually, the TOP method is not a genomic prediction (GS) algorithm that focus on the accurately predict trait values. Given most GS methods focus on the prediction trait by trait. Thus, our method aimed to integrate multi-trait predictions for decision making of individual selection especially for the traits are nonsynergistically correlated. As the reviewer#2 commented, our method is different to traditional GS methods (including multi-trait GS). However, our TOP method is more like the breeding selection approach such as culling selection and index selection when multiple trait values are available from either measured or predicted. In our research, based on the real data (Figure 3b), we compared the performance of TOP method and other selection methods. We* *found that TOP method outperformed the culling and index selection methods when simultaneously considering more than two traits in realistic breeding scenarios.*

Comment 5: A step by step protocol of TOP in analyzing the dataset should be provided in Supplementary Text.

*Our response: As suggested by the reviewer, we have provided a detailed tutorial text for step-by-step protocol of TOP in the Additional file 2: TOP tutorial and it can also be accessed at the GitHub (https://github.com/yingjiexiao/TOP).*

Reviewer 2#:

The article "Targeted selection by integrating organismal and molecular traits through predictive analytics" by Yang et al introduces and demonstrates a new multi-trait ML indexing approach for assessing and predicting the "similarity" of breeding lines against a commercial check, or ideotype.

They demonstrate that their tool is able to increase the proportion of elite candidates within the selection thresholds for target traits, while not depleting the value of other traits.

The authors provide all code and demo examples on GITHUB, which run on R version 4.1.0 (2021-05-18) Platform: x86_64-apple-darwin17.0 (64-bit) Running under: macOS 12.1.

This paper is well written and easy to understand because the objective was clear from the beginning, which is appreciated.

*Our response: We are very grateful for the reviewer’s nice comments.*

If I understand correctly (please correct me if I am wrong), the strategy is to use GBLUP for t traits (1 trait at a time) and then to make selections based on the 'superiority' of target trait t_1 among hybrids that have a high predicted similarity among target traits t_2,...,t_i. So in a way this is independent culling where the first cull trait is a selection index built from second/third priority traits?

*Our response: The reviewer has correctly understanded our strategy of the TOP method. It included two main steps: i) obtain predicted values for t traits one by one using GBLUP (not limited to it); ii) based on predicted traits make selection for hybrids that are superior to the target variety for trait 1 while have a high similarity to the target for traits 2, … , t. As the reviewer mentioned, the similarity function (step#ii) can be recognized as a machine learning based index approach which optimize the trait weights by simultaneously considering the integrity and balance relations of multiple traits. However, to technically perform multi-trait selection, our method chose to build a full similarity function for all t traits (the first trait and other t-1 traits). We modified the first trait of the target variety (e.g. reduced 5% flowering time, or increased 5% yield) to build an improved target, that may not really exist but is literally what we want to improve the trait. The other traits of the improved target were kept the same to the original target. The similarity search to the improved target will help us increase the proportion of elite hybrids that are superior to specific trait while relatively stable to other traits. So our method is technically not an independent culling approach or combination approach of culling and index way. But the logics of these methods are highly similar, I feel it deserve to test their performances in our following work.*

I do have mostly minor comments and questions.

*Our response: Thank you for the valuable comments for improving our work.*

Comment 1: In general, the approach taken is to target similarity to some existing hybrid, e.g., Zhengdan958, Xianyu335, and Jingke968. I think this is creative, and allows researchers to take advantage of existing -omics data. However, I do not know how these three lines (especially Zhengdan958) compare to the rest of the population in terms of target traits. Figs 5 and 6, lead me to believe that Zhengdan958 must be mediocre in with respect to days to tassel and plant height. I understand that this hybrid is a popular cultivar, but I wonder if this example over emphasizes the performance of TOP? As an example, do you know what happens when yield is the target trait and the commercial check is in the upper tail of the distribution?

*Our response: The reviewer is right that the target position will influence the TOP performance. Using days to tassel (flowering time) and plant height as example to illustrate, early flowering and short plant height are breeding valuable trait direction for stress avoidance and logging resistance. The flowering time of Zhengdan958 indeed located in the middle of the whole population, which can be seen that the proportion of random selected hybrids flowered earlier or later than Zhengdan958 is 50% (Fig. 5a); but the plant height of Zhengdan958 located in the lower tail of the population distribution, which can be seen that the proportion of random selected hybrids were shorter and taller than Zhengdan958 is 16% and 84% (Fig. S5a). For flowering time (middle positioned), our method improved the success selection by ~2 folds compared to by chance (89% vs 50% for early hybrids, 98% vs 50% for late hybrids) (Fig. 5a). For plant height (lower-tail positioned), our method can have a marginal selection improvement than random by ~1.2 fold for taller hybrids, but achieve to ~4.4 fold for short hybrids. These results demonstrate our method had high ability to assist trait improvement compared to by chance, especially for the extreme target, because the extreme target should reduce the successful selection rate in any approaches, but the extreme situation may further highlight the usefulness of our method in enrichment of elite candidate hybrids.*

Specifically, I am curious how TOP performs when my ideotype is extreme for the primary target trait (ear weight). Perhaps this is what is being shown in Fig S6, S7, S8, and S10. However, Zhengdan958 "line" in S6, S7, S8 vid S10 seems to be in different places. from the first three, I think Zhengdan958 has g/ear = ~248, but in S10 is it plotted at 215 g/ear? maybe I am reading this wrong.

*Our response: As we explained above, we think TOP performed better when the target is extreme for the primary trait. In the extreme case, all selection approach should have low successful selection rate (e.g., the proportion of shorter hybrids than the target), but our method can largely enrich the elite hybrids that increased the probability of selecting elite hybrids. To better explore it, we additional chose four hybrids as targets, located at the 10th, 20th, 80th and 90th percentile of the distribution of flowering time. We found similar results that the extremer the target is, the absolute success selection rate goes lower but TOP improvement goes higher (Figure S7).*

*We apologized for the misleading of Zhengdan958’s ear weight. The two values are correct but different, because ~248 g/ear is the best linear unbiased predictor (BLUP) value across five environments in 2015 for testing TOP method, while 215 g/ear is the plot average value for validation field trial in Hainan province of China in 2019 for independent validation. The difference may be due to environment effect.*

Comment 2: How would I use this in the case where my 'ideotype' is unobserved in my population?

*Our response: Interesting comment. Strictly saying, the results for selecting hybrids that outperform an existing commercial variety should be the case, that the ideotype is the improved version of an existing hybrids and may not realistically exist. Generally, in contrast to base on an existing variety, the breeders can de novo design a series of trait values as an ideotype based on breeding experiences and multi-discipline progress such as agronomy, plant physiology and crop growth model. Our method has the potential to recommend optimal individuals for approaching the designed ideotype. It’s interesting and challenging and deserve to be explored further.*

Comment 3: Figure 2 only has A and B labels, but caption has A-D.

*Our response: We apologized for that mistake. We have corrected the labels.*

Comment 4: I feel that the multi trait GS references are a bit out of date and makes it seem that there has been limited improvement on multi-trait prediction, when in reality this is a rapidly growing space. I was able to find several more recent references with a quick google scholar search, e.g., Runcie et al 2021 - MegaLMM and Montesinos-López et al 2021 - multi-year wheat breeding trials. These approaches are different that yours, but are still part of this space.

*Our response: Thank you for the reviewer’s kind suggestion. We had discussed more on the progress of multi-trait predictions in the discussion. The state-of-art of references were added. As the reviewer mentioned, in this paper, the GS part is not the core of the TOP method that focused on integrating multiple traits for making selection. This paper used GBLUP (a single trait prediction method) to demonstrate the utility of TOP method, but we agree the progress of multi-trait prediction method are fast, such as MegaLMM, it can leverage thousands of traits at once to enhance prediction accuracy. We believe any state-of-art single-trait or multi-trait GS methods are complementary to our proposed TOP method. They can be used for obtain highly accurate trait predictions, which will be beneficial to boost selection efficiency by TOP method.*

Comment 5: "the similarity function" Line 414-416 are redundant with 404-406 and I think there are missing super/sub scripts in 414-416. "O" = observed and "Y" = predicted is non-standard and I would suggest using "Y" = observed and "Y_hat" = predicted.

I do not think that "N" or "d" are defined in this section? I believe that they are population size and trait number, respectively. Please make sure that all variables are defined as they appear in text.

*Our response: Thank you for the suggestions. We have removed the redundant parts, changed the corresponding symbols, and added the definition of variables.*

*The similarity function was expressed as:*

*P(Y^n,Y ̂^n)=(exp⁡(-∑_(i=1)^d▒〖w_i |Y_i^n-Y ̂_i^n | 〗))/(∑_(n=1)^N▒〖exp⁡(-∑_(i=1)^d▒〖w_i |Y_i^n-Y ̂_i^n | 〗)〗), where Y_i^n and Y ̂_i^n are observed and predicted values, respectively, of the trait i in the individual n, d is trait number, N is the training population size, and w_i is the weight for the trait i. For omics data, traits here refer to the principal components that retain most (>80%) of the variation presented in the original transcriptomic or metabolic features.*

*The optimal weights of multiple traits were obtained by maximizing the likelihood function L(w)=∏_(n=1)^N▒〖P(Y^n,Y ̂^n)〗. This maximizes the following function: ln⁡(L(w))=∑_(n=1)^N▒〖lnP(Y^n,Y ̂^n)〗 with respect to the weights.*

*In the testing population, the similarity degree was defined by the above similarity function in the training population, denoted by P(Y,Y ̂^n),*

*where Y is the observed value of a given target; Y ̂^n is the predicted value of the individual n in the testing population.*

Comment 6: How stochastic is the similarity matrix, i.e., similarity_matrix, in 'TOP_demo.r'? I ran the demo multiple times and got different results each time despite no changes to the code or the test/train set, etc. I suppose the stochasticity is from the individuals in the pools? I think that this needs more explanation in the code documentation.

> (similarity_matrix=ide_rate$Demo_p)

[,1] [,2] [,3] [,4] [,5]

[1,] 0.47245376 0.10275327 0.01271944 0.34940391 0.06266962

[2,] 0.04889945 0.33186839 0.03028727 0.04481262 0.54413227

[3,] 0.08440274 0.02529848 0.69900723 0.13965996 0.05163159

[4,] 0.12692922 0.03706160 0.01440462 0.74838112 0.07322343

[5,] 0.05898381 0.35695537 0.03075991 0.03849533 0.51480558

> (similarity_matrix=ide_rate$Demo_p)

[,1] [,2] [,3] [,4] [,5]

[1,] 0.55464817 0.125228438 0.187359011 0.12249032 0.010274055

[2,] 0.01311712 0.753341013 0.206353781 0.02692470 0.000263382

[3,] 0.03136113 0.543135510 0.399058223 0.02532194 0.001123195

[4,] 0.30236444 0.033900282 0.052966046 0.55701789 0.053751344

[5,] 0.06667338 0.002611329 0.009516418 0.04602473 0.875174149

Also, I am confused as to how I would use `Test_top_res()` to specify a specific target, e.g., Zhengdan958?

I am not entirely sure how to interpret the demo results and I think it would be beneficial to increase the amount of documentation so that a complete novice could work with and interpret the code you have compiled.

It might also be helpful to build a demo where there is 1 specific target ideotype selected from the dataset.

*Our response: We apologized for the unclear description. The reviewer is right that the stochasticity is from the individuals in the pools. We have added random seeds to make sure that the output of similarity matrix is reproducible. Following the reviewer’s kind suggestion, we had rewritten the tutorial of our TOP method including input, usage and output sections. The input section used the snapshot of data format, R functions and detailed descriptions to show which kind of data is needed for TOP method, and how to be read into R analytic platform. The usage section showed how to perform trait predictions, optimal weight learning, selection for elite individuals to a specified target. The output section step-by-step interpreted the analytic results, such as optimal weight, similarity matrix, selection individuals and basic visualizations. We hope the current tutorial may be helpful for the novice peers or related experts to run our TOP method and get valuable implications for breeding work. The tutorial is provided as the Additional file 2: TOP tutorial and stored at the GitHub (https://github.com/yingjiexiao/TOP). We will continue updating the TOP method and improving the tutorial, which can be seen in the GitHub access.*

Comment 7: "The transcriptomic and metabolic variables were composed into 88 and 24 traits, respectively, via principal component analysis (PCA)." What lead to this decision? Scree plot? variance explained? What is "the primary variation of the original transcriptomic or metabolic features."?

*Our response: We apologized for the misleading. In our study, we chose the cumulative variance of 80% as the threshold to keep few principal components that can retain most of the variation presented in all original variables. We have revised the relevant sentence in the method for clarity in the revised manuscript.*

**Second round of review**

**Reviewer 1**

I'm satisfied with the revision.

Most of the concerns from Reviewer#2 have been well addressed. Only two remaining questions should be clarified.

1. The tool can work well by using CUBIC data, which was developed by inter-crossing 24 lines (Genome Biol. 2020, 21:20) and further crossing with 30 testers (Genome Biol. 2021, 22:148). However, the 54 lines cannot cover the full diversity in maize breeding (e.g., Xianyu335 not included), and the datasets like CUBIC (genotypes and phenotypes of 1,428 inbred lines plus 8,652 hybrid lines) are fewly available in publical databases. Since the tool strongly relies high-quality panel, the utility of the tool, as well as matters needing attention, should be discussed.

2. Why not using multi-trait prediction, instead of single-trait GBLUP? It could be discussed.

**Authors Response**

**Point-by-point responses to the reviewers’ comments:**

Reviewer 1#:

I'm satisfied with the revision.

Most of the concerns from Reviewer#2 have been well addressed. Only two remaining questions should be clarified.

1. The tool can work well by using CUBIC data, which was developed by inter-crossing 24 lines (Genome Biol. 2020, 21:20) and further crossing with 30 testers (Genome Biol. 2021, 22:148). However, the 54 lines cannot cover the full diversity in maize breeding (e.g., Xianyu335 not included), and the datasets like CUBIC (genotypes and phenotypes of 1,428 inbred lines plus 8,652 hybrid lines) are fewly available in publical databases. Since the tool strongly relies high-quality panel, the utility of the tool, as well as matters needing attention, should be discussed.

*Response: Thank you for your suggestions. We acknowledge that the 54 founders of CUBIC-based F1 population did not cover full diversity in maize breeding. But we want to say that, as single experiments of CUBIC and NCII populations, the best efforts had been made trying cover the most breeding diversity by including 24 elite inbred lines mostly used in past two decades of China breeding and 30 independent elite lines derived from five heterotic groups. The huge amount of work for the CUBIC initiative started from 2004 by developing the population and continued nearly 10 years. We feel sorry that the original link of genotype and phenotypes of inbreds and hybrids listed in the Availability of Data and Materials in our previous publication (Genome Biol, 2021, 22:148) is invalid due to disk problem. The datasets are now publicly available in China National GeneBank (https://ftp.cngb.org/pub/CNSA/data3/CNP0001565/zeamap/99_MaizegoResources/01_CUBIC_related/).*

*To the utility of TOP method, we found it can work universally well in CUBIC data, two independent maize diverse populations (368 inbred lines and 282 inbred lines) and one rice RIL population (210 lines) as demonstrated in the paper. One advantage of TOP method is to help select individuals superior to a target variety. The current algorithm is to search the similarity of all traits to an improved target where one trait was modified while others were kept the same. In the future, we will test the performance by combining TOP and culling selection, where one trait was culled after TOP selection on other traits was done. another advantaged is that the strength of TOP is enhanced when omics level data are included, which will be urgent in this era of biological big data. We however need to be cautious that significant amount of omics data may be biased if included when they are barely controlled by genetics. We discussed this limitation and future perspective of TOP method in the revised manuscript.*

2. Why not using multi-trait prediction, instead of single-trait GBLUP? It could be discussed.

Response: Thank you for the suggestion. As previously commented by the reviewer#2, different from multi-trait prediction methods (e.g., MegaLMM) for predicting trait values, our proposed TOP method is for integrating predicted values of multiple traits to identify an ideotype individual via learning balanced relations among traits. To this aim, TOP method is for selection decision after trait values is predicted. The section of trait prediction is out of the scope of current TOP method, so we choose the most recognizable GS method in plant breeding, GBLUP, which is a robust single-trait prediction approach. We however agree that multi-trait GS methods may further enhance the TOP performance if multi-trait GS methods are accurately than the single-trait for trait prediction. We had discussed this issue that “As TOP is an integrative strategy that employs genomic prediction, omics, phenomics, and machine learning, improvement of these technologies are also expected to boost TOP performance” in the revised manuscript.

*Response: Thank you for the suggestion. As previously commented by the reviewer#2, different from multi-trait prediction methods (e.g., MegaLMM) for predicting trait values, our proposed TOP method is for integrating predicted values of multiple traits to identify an ideotype individual via learning balanced relations among traits. To this aim, TOP method is for selection decision after trait values is predicted. The section of trait prediction is out of the scope of current TOP method, so we choose the most recognizable GS method in plant breeding, GBLUP, which is a robust single-trait prediction approach. We however agree that multi-trait GS methods may further enhance the TOP performance if multi-trait GS methods are accurately than the single-trait for trait prediction. We had discussed this issue that “As TOP is an integrative strategy that employs genomic prediction, omics, phenomics, and machine learning, improvement of these technologies are also expected to boost TOP performance” in the revised manuscript.*
